# Water Inside the Carbon Cage—H_2_O@C_60_ and Related Fullerene Structures: Synthesis, Experimental Characterization, Computational Insights, and Future Perspectives

**DOI:** 10.3390/ijms27198835

**Published:** 2026-10-03

**Authors:** Monika Zielińska-Pisklak, Zuzanna Kuś, Marcin Gackowski, Łukasz Szeleszczuk

**Affiliations:** 1 Department of Pharmaceutical Chemistry and Biomaterials, Faculty of Pharmacy, Medical University of Warsaw, 1 Banacha Str., 02-093 Warsaw, Poland; 2 Department of Organic and Physical Chemistry, Medical University of Warsaw, 1 Banacha Str., 02-097 Warsaw, Poland; s088894@student.wum.edu.pl (Z.K.); lukasz.szeleszczuk@wum.edu.pl (Ł.S.); 3 Department of Toxicology and Bromatology, Faculty of Pharmacy, L. Rydygier Collegium Medicum in Bydgoszcz, Nicolaus Copernicus University in Torun, 2 Jurasza Str., 85-089 Bydgoszcz, Poland; marcin.gackowski@cm.umk.pl

**Keywords:** H_2_O@C_60_, water endofullerenes, endohedral fullerenes, molecular surgery, nanoconfinement, quantum molecular dynamics, nuclear-spin isomerism, host–guest interactions

## Abstract

The encapsulation of a single water molecule inside C_60_ has established H_2_O@C_60_ as a benchmark system for investigating molecular behavior under precisely defined subnanometer confinement. This review integrates advances in the synthesis, experimental characterization, theoretical description, and emerging applications of H_2_O@C_60_ and selected related water-containing fullerenes. Particular attention is given to molecular-surgery strategies, in which the fullerene cage is opened, loaded with water, and subsequently reconstructed, as well as to improvements that have increased encapsulation and cage-closure efficiencies and enabled isotopically labeled and heterofullerene derivatives. Structural, nuclear magnetic resonance, infrared, terahertz, neutron-scattering, dielectric, photoelectron, and single-molecule transport studies collectively show that the C_60_ framework remains highly rigid, while the confined water molecule retains pronounced translational, rotational, vibrational, and nuclear-spin dynamics. At the same time, weak but measurable host–guest interactions produce symmetry breaking, rotational-level splitting, electrostatic screening, modified vibrational responses, and environment-dependent ortho–para conversion. Computational studies, ranging from density functional and correlated wave-function methods to multidimensional quantum-dynamical and molecular-dynamics approaches, have been essential for interpreting these observations and defining the underlying potential-energy landscape. Related systems, including H_2_O@C_70_, H_2_O@C_59_N, charged azafullerenes, and isotopically substituted endofullerenes, provide routes to tune cage geometry and electrostatic interactions. Although technological applications remain largely at the proof-of-principle stage, these systems offer promising platforms for quantum spectroscopy, molecular electronics, and the rational design of functional host–guest nanomaterials.

## 1. Introduction

### 1.1. General Overview of Fullerene Chemistry and Encapsulation Phenomena

Since their discovery in 1985, carbon fullerenes have evolved from objects of fundamental scientific interest into an important class of molecular nanomaterials used in physics, chemistry, and materials science [1,2]. The prototypical fullerene, buckminsterfullerene C_60_, adopts a nearly spherical truncated-icosahedral structure built from 12 pentagons and 20 hexagons arranged within a conjugated π-electron framework [3,4]. This structural motif accounts for the unusual combination of high thermodynamic and kinetic stability, well-defined redox properties, characteristic photophysical behavior, and broad chemical reactivity that has made C_60_ one of the central model systems in carbon nanoscience [5,6].

The chemistry of fullerenes includes a wide range of covalent and noncovalent transformations, from classical cycloaddition and hydrogenation reactions to redox-controlled functionalization, supramolecular binding, and heteroatom incorporation into the carbon framework [3,7,8,9,10]. Their reactivity is strongly influenced by the intrinsic curvature of the cage and by the unequal distribution of strain across 6–6 and 5–6 ring junctions, which govern regioselective addition patterns and enable controlled modification of the carbon skeleton [3,11,12]. These structural features have been extensively exploited to prepare structurally diverse fullerene derivatives with tunable optoelectronic, magnetic, and interfacial properties, including organofullerenes, fulleropyrrolidines, and donor–acceptor architectures [8,13,14]. One of the most important areas of fullerene chemistry is the preparation of endohedral fullerene complexes, in which guest species are encapsulated within carbon nanocages to form well-defined host–guest systems for fundamental and applied research [3,4,5,6,7,8,9,10].

A distinctive feature of fullerenes is the presence of an internal cavity that enables them to host atoms, ions, or small molecules either inside the cage, as endohedral complexes, or at the outer surface through exohedral interactions [6,15]. Endohedral fullerenes (X@C_n_) constitute a distinct class of molecular systems in which an atom or small species is confined within an intact carbon cage, while the electronic interaction with the π-conjugated host is generally limited and highly system-dependent [6,16]. The extent of host–guest interaction is governed by the size and symmetry of the carbon cage, the chemical nature of the encapsulated species, and the geometric constraints imposed by cage curvature [17,18,19]. Encapsulation can stabilize otherwise highly reactive atoms, ions, or small molecules, while altering their spin states, charge distribution, and magnetic behavior, giving rise to well-defined quantum-confined systems of interest for molecular electronics, spin-based technologies, and quantum information science [18,20].

The encapsulation of small neutral molecules—such as H_2_, H_2_O, NH_3_, CO, and CH_4_—within fullerene-based systems, either as closed endohedral species or via open-cage intermediates, has developed into a well-established research field because of the interplay between molecular motion and nanoscale confinement [21,22,23,24,25,26,27,28,29,30,31]. The internal cavity of C_60_, with a diameter of approximately 3.5–3.7 Å [32,33], is too small to allow spontaneous diffusion of neutral molecules through the intact carbon framework. As a result, encapsulation must rely on non-equilibrium synthetic strategies, most notably molecular-surgery protocols involving stepwise cage opening, guest insertion, and re-closure, as well as high-energy approaches such as ion implantation or arc-discharge-based formation [15,34,35,36]. These methods provide access to well-defined endohedral complexes in which the guest is isolated from the external environment, thereby enabling experimental investigation of rotational tunneling, quantum dynamics, and guest–host interactions under precisely defined confinement conditions.

**Figure 1:**
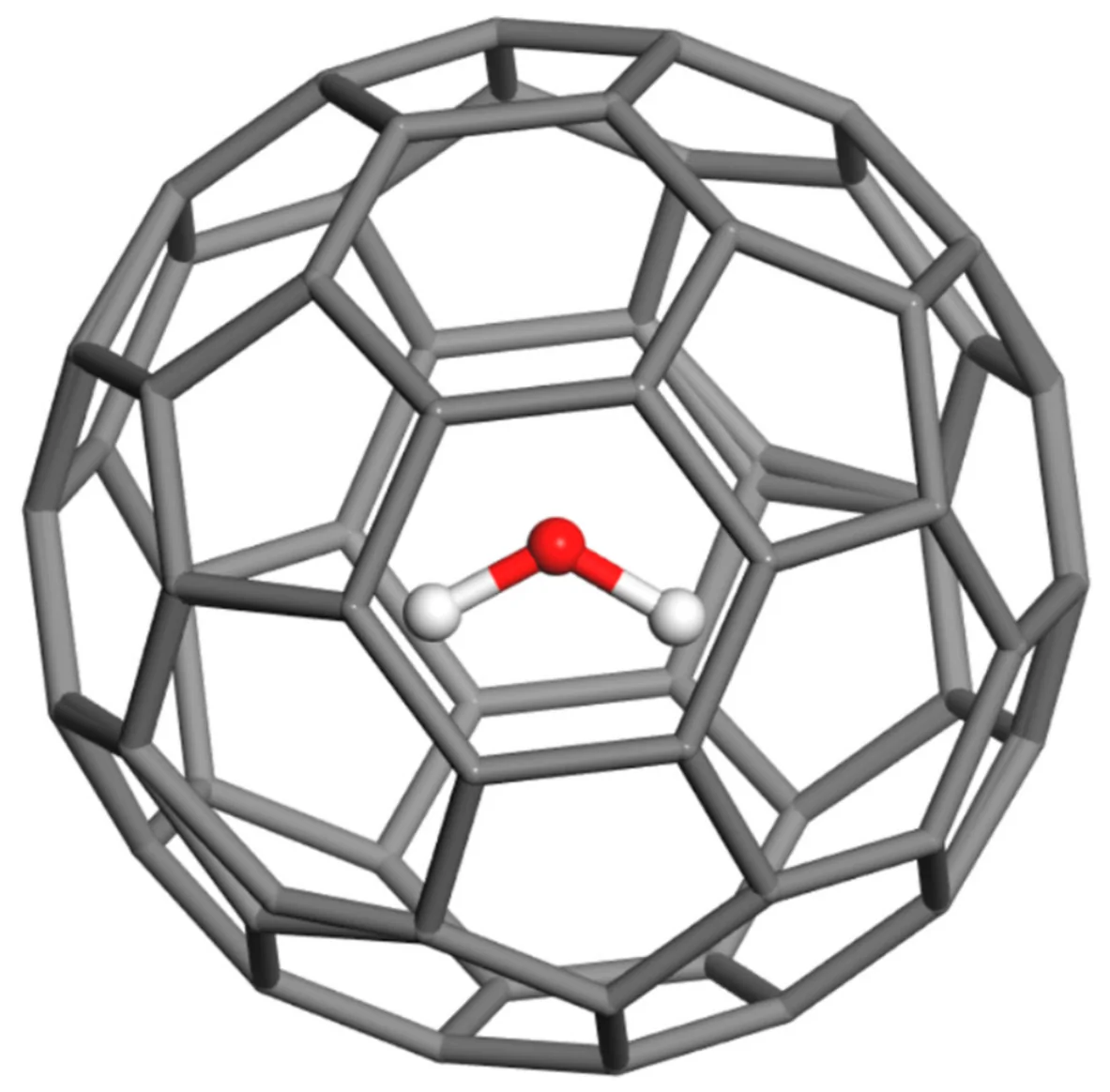
Molecular architecture of H_2_O@C_60_ based on the crystallographic structure deposited in the Cambridge Structural Database (CSD refcode: BIMGIP), reported by Kurotobi and Murata [36].

The successful encapsulation of a single water molecule inside C_60_ marked a major milestone in molecular endofullerene chemistry. Among all molecular endofullerenes investigated to date, H_2_O@C_60_ occupies a particularly distinctive position (Figure 1). Unlike noble gases or non-polar guest molecules, water possesses a permanent electric dipole moment, rich rotational, translational, and vibrational dynamics, and nuclear-spin isomerism, making it a sensitive molecular probe of confinement effects and host–guest interactions [22,36,37,38,39,40,41,42]. The isolation of a single H_2_O molecule within a structurally well-defined hydrophobic cavity created an unprecedented model system for investigating the intrinsic behavior of water in the absence of bulk and interfacial effects, while eliminating the complexity associated with the intermolecular hydrogen-bonding network [22,36,43]. H_2_O@C_60_ offers rare experimental access to quantum rotational states, ortho–para spin isomerism, dielectric response, and vibrational properties modified by confinement within a precisely defined nanoscale environment [37,38,39,40,44]. Moreover, the encapsulated water molecule serves as a sensitive probe of cage symmetry and guest–host interactions, as demonstrated by complementary NMR, dielectric, infrared, terahertz, and computational studies [37,38,39,41]. Consequently, H_2_O@C_60_ has become one of the best-characterized benchmark systems for correlating experimental observations with high-level theoretical descriptions of molecular confinement [36,37,42,45].

The study of molecules confined within fullerene-based systems thus lies at the intersection of synthetic chemistry, spectroscopy, quantum dynamics, computational modeling, and materials science [42,46,47,48,49]. In this context, X@C_60_ systems—and in particular H_2_O@C_60_—provide well-defined molecular platforms for examining how sub-nanometer confinement influences structural, electronic, spectroscopic, and dynamical behavior. Insights obtained from these systems extend beyond fundamental chemical physics to emerging areas including molecular electronics, spin-dependent phenomena, quantum information science, and the rational design of nanoscale host–guest architecture [37,38,39,44].

While previous reviews have addressed endohedral fullerene chemistry, molecular-surgery methodologies, or selected spectroscopic aspects of non-metallic endofullerenes [6,31,42], the present review is intended to provide more than a combined summary of these areas. Its central aim is to critically correlate synthetic strategy, molecular structure, spectroscopic response, quantum dynamics, and computational description within a unified framework focused on H_2_O@C_60_ and related water-containing fullerene systems. Particular attention is given to how variations in cage geometry, charge, external environment, and host–guest interactions are reflected across diffraction, NMR, vibrational, neutron-scattering, dielectric, photoelectron, and single-molecule measurements, and how these observations are reproduced or rationalized by theoretical models. The review also explicitly considers apparently different structural descriptions of confined water, including average near-central positions and environment-induced off-center displacement or effective symmetry breaking, and discusses how these observations can be reconciled. Finally, experimentally demonstrated properties are distinguished from more speculative applications in molecular electronics, quantum-state control, and spin-based technologies. In this way, the review aims to provide a critical cross-comparison of experimental and theoretical evidence rather than a purely chronological or technique-by-technique survey of the field.

The primary focus of this review is H_2_O@C_60_, for which the most extensive body of synthetic, structural, spectroscopic, and theoretical information is currently available. Related water-containing fullerene systems are discussed selectively where they provide a meaningful comparison with this benchmark. In particular, H_2_O@C_70_ is considered to illustrate the consequences of increased cage size and intrinsic geometric anisotropy, whereas H_2_O@C_59_N and H_2_O@C_59_N^+^ provide examples of how heteroatom substitution and cage charge modify the local electrostatic environment and host–guest interactions. Isotopically substituted systems, including D_2_O@C_60_ and H_2_^17^O@C_60_, are included where they provide complementary information on molecular dynamics, nuclear-spin behavior, or spectroscopic assignment. Theoretical models involving multiple confined water molecules are discussed separately as extensions of the single-molecule confinement problem and are not treated as equivalent to experimentally established H_2_O@C_60_-based endofullerenes.

### 1.2. Evolution of Endohedral Fullerene Chemistry: From Metallofullerenes to Molecular Endofullerenes

The development of endohedral fullerene chemistry closely followed the discovery and subsequent bulk production of C_60_, which made fullerene materials available for systematic isolation and spectroscopic characterization [1,2]. Early cluster-beam mass-spectrometric experiments provided evidence for lanthanum-containing fullerene species, including LaC_60_, whereas subsequent arc-discharge synthesis, chromatographic separation, and structural studies established solvent-extractable metallofullerenes such as La@C_82_ and related M@C_n_ systems [6,15,50]. These findings demonstrated that metal atoms could become incorporated during fullerene formation and remain stably confined within the resulting carbon cages. In many endohedral metallofullerenes, substantial metal-to-cage electron transfer, together with system-dependent ionic and covalent contributions, strongly modifies the electronic, magnetic, and redox properties of both the guest and the carbon framework [6,15,50].

During the 1990s and early 2000s, advances in chromatographic separation, NMR, electron-spin resonance, optical spectroscopy, electrochemistry, and crystallography enabled increasingly detailed characterization of monometallic, multimetallic, and cluster endofullerenes [6,50]. In parallel, non-metallic atomic endofullerenes were obtained through high-energy collision or implantation experiments and by high-pressure, high-temperature loading of preformed fullerene cages. Noble-gas endofullerenes and spin-active species such as N@C_60_ and P@C_60_ demonstrated that fullerene cages can also stabilize chemically inert or weakly interacting guests without conventional covalent bonding to the carbon framework [6]. These systems broadened endohedral chemistry beyond metallofullerenes and, for selected spin-active species, stimulated interest in molecular-spin and quantum-information technologies [6,50].

Despite these advances, the controlled encapsulation of predefined neutral molecules remained a major synthetic challenge. Unlike metal atoms, which may become trapped during cage formation, molecular guests generally cannot be incorporated efficiently by conventional arc-discharge, ion-implantation, or high-pressure methods. This limitation motivated the development of chemical strategies in which the fullerene cage is temporarily opened, loaded with a selected guest, and subsequently restored [6,31,34]. The molecular-surgery concept emerged from pioneering cage-opening studies by Wudl, Rubin, and co-workers and was subsequently developed into complete synthetic routes to closed molecular endofullerenes by the groups of Komatsu, Murata, and others [31,34,51,52,53,54,55]. These procedures use carefully designed sequences of organic transformations to enlarge a cage orifice, introduce the guest, and reconstruct the intact fullerene framework, thereby offering precise control over the identity of the encapsulated species [23,31,34,56].

A decisive proof of concept was the organic synthesis of closed H_2_@C_60_, culminating in the 2005 report by Komatsu, Murata, and co-workers [23,31,34]. The resulting material was a chemically stable and well-defined molecular endofullerene that could be isolated in preparative quantities and subsequently handled under ambient conditions. Its availability enabled detailed investigations of quantized guest motion inside an intact carbon cage and established the conceptual and experimental foundation for the encapsulation of more complex neutral molecules [23,31,34,57].

Building on these achievements, Kurotobi and Murata reported the first synthesis of H_2_O@C_60_ in 2011, representing a major transition from atomic and non-polar molecular guests to a confined polar polyatomic molecule [36]. Water combines a permanent electric dipole moment, internal rotational and vibrational degrees of freedom, and ortho and para nuclear-spin isomers. Its encapsulation therefore created a particularly informative model system for investigating how a highly symmetric carbon nanocage modifies molecular motion, electrostatic response, and nuclear-spin behavior while preventing intermolecular hydrogen bonding. Subsequent experimental and theoretical investigations of H_2_O@C_60_, together with studies of other molecular and atomic endofullerenes, greatly expanded our understanding of guest–host interactions and molecular confinement. NMR, infrared, terahertz, neutron-scattering, dielectric, and computational studies revealed phenomena including nearly free but weakly perturbed quantum rotation, confinement-induced splitting of rotational states, dipole screening, ortho–para conversion, and subtle guest–host coupling, demonstrating the richness of molecular behavior inside fullerene cages [22,36,37,40,41,42,56,58,59,60,61,62,63,64,65].

Collectively, these studies established H_2_O@C_60_ as a benchmark system for investigating molecular confinement and guest–host interactions. The remarkable expansion of this field over the past decade provides the motivation for the present review, which summarizes recent advances in the synthesis, experimental characterization, computational investigations, and emerging applications of H_2_O@C_60_ and related fullerene systems.

## 2. Strategies for the Preparation of H_2_O@C_60_ and Related Fullerene Structures

**Figure 2:**
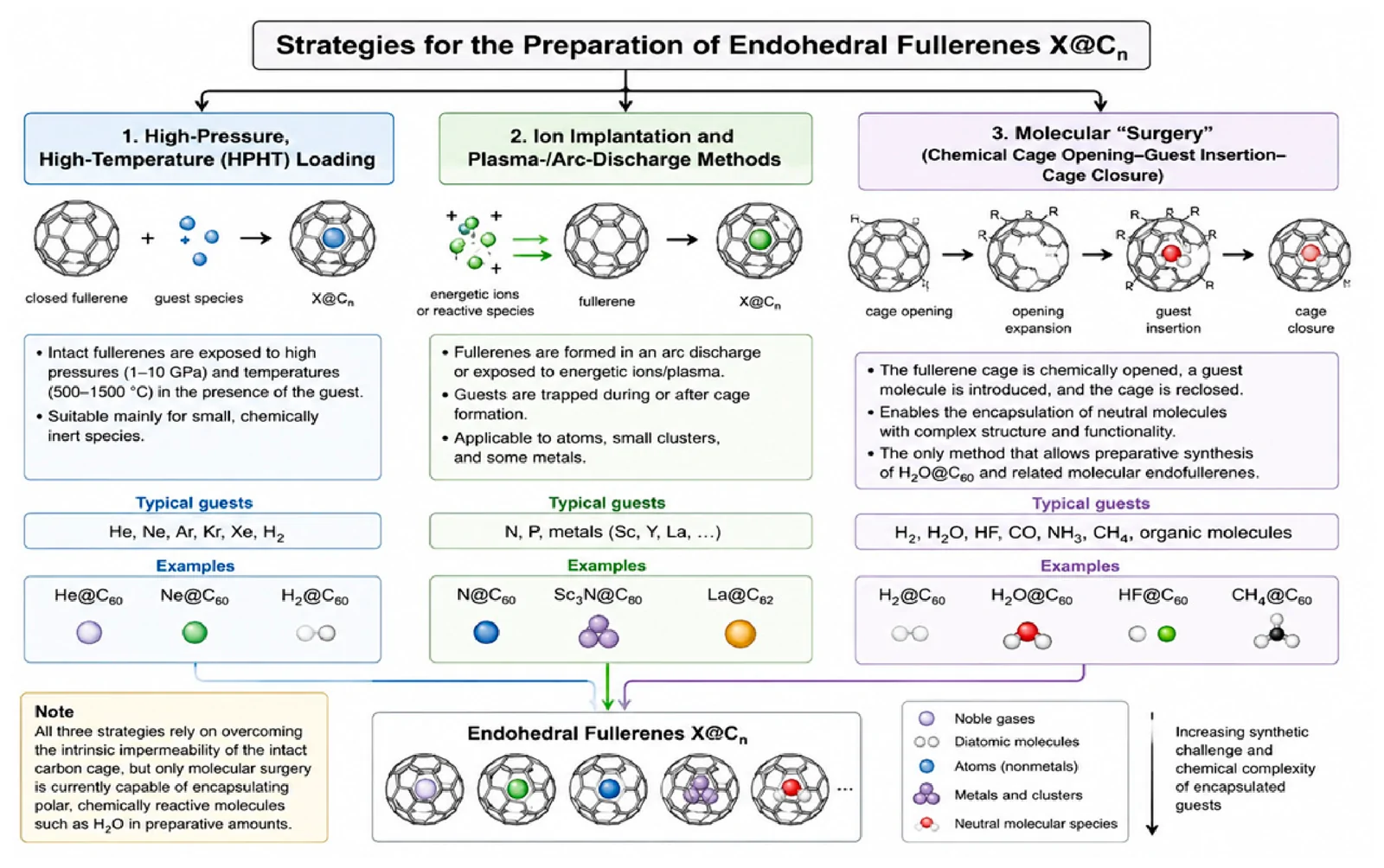
Principal synthetic strategies for the preparation of endohedral fullerenes (X@C_n_), including high-pressure/high-temperature (HPHT) loading, ion implantation/plasma- or arc-discharge methods, and molecular surgery. Figure was created using OpenAI ChatGPT version 5.6 Sol.

The preparation of endohedral fullerenes X@C_n_ requires synthetic and physicochemical approaches capable of overcoming the intrinsic impermeability of the intact carbon cage. Over the past three decades, three principal approaches have been developed: (i) high-pressure, high-temperature (HPHT) loading of closed fullerenes; (ii) ion implantation and plasma- or arc-discharge methods, which exploit energetic species during or after fullerene formation; and (iii) chemical “molecular surgery”, in which the fullerene cage is temporarily opened, a guest molecule is inserted, and the carbon framework is subsequently reclosed. Although all three strategies have contributed to the development of endohedral fullerene chemistry, only molecular-surgery methodologies have enabled the routine preparation of neutral molecular endofullerenes such as H_2_O@C_60_ in preparative quantities (Figure 2). Consequently, the historical evolution of these approaches reflects the progressive search for synthetic routes capable of encapsulating increasingly complex molecular guests. Comprehensive reviews describing these methodologies have been published for endohedral fullerenes in general and endohedral metallofullerenes in particular [6,34,50].

Among neutral molecular guests, water proved to be one of the most challenging targets for encapsulation because it could not be introduced efficiently using available physical encapsulation methods, requiring the development of dedicated molecular-surgery methodologies. Whereas HPHT loading and ion implantation proved successful primarily for noble gases and selected atomic guests, the preparation of H_2_O@C_60_ has relied almost exclusively on molecular-surgery methodologies. Nevertheless, the earlier physical encapsulation strategies played an essential role in demonstrating that intact fullerene cages can accommodate encapsulated species and therefore established the conceptual foundation for the subsequent development of molecular endofullerenes containing neutral molecules [6,23,34,36].

### 2.1. High-Pressure, High-Temperature (HPHT) Methods

The first successful preparation of non-metal endohedral fullerenes was achieved by exposing solid C_60_ to noble gases under high-pressure, high-temperature (HPHT) conditions, allowing atoms to diffuse through the carbon cage under strongly nonequilibrium conditions. In a seminal study, Saunders and co-workers demonstrated the formation of He@C_60_ and Ne@C_60_ by heating C_60_ in helium or neon at several kilobars and temperatures of approximately 600–650 °C, giving low but measurable fractions of endohedral species [56,58]. These pioneering experiments provided the first convincing evidence that intact fullerene cages are not completely impermeable under extreme thermodynamic conditions, thereby establishing the conceptual basis for subsequent encapsulation strategies. The methodology was later extended to Ar, Kr, and Xe, establishing a family of noble-gas endofullerenes, including Ng@C_60_ and Ng@C_70_ [6,59,60].

HPHT loading typically yields very low encapsulation fractions (often 10^−6^–10^−3^) of the fullerene sample, reflecting the high activation barrier for penetration through the closed carbon cage, and competing cage damage and fragmentation processes [6,56,58,59,60]. Selective chromatographic separation and ^3^He NMR spectroscopy are therefore generally required to isolate and characterize sufficient quantities of endohedral products [6,61]. The method is particularly well suited to small, chemically inert atoms (He, Ne, Ar, Kr, Xe), whereas larger or chemically reactive species are generally not encapsulated efficiently under these conditions [6,56,58,59,60].

An important modification of HPHT loading is the explosive or shock-compression method, in which an explosive is detonated in a closed vessel containing C_60_ and a noble gas. Peng and co-workers demonstrated that the transient increase in gas kinetic energy markedly enhances the probability of penetration through the fullerene cage, enabling the preparation of He@C_60_ and He_2_@C_60_ in significantly higher yields than those achieved by conventional static HPHT loading [58,62]. Nevertheless, this approach also remains largely restricted to small atomic guests.

From the perspective of H_2_O@C_60_, however, HPHT and related physical loading methods have proved unsuccessful. Unlike noble gases, water could not be efficiently encapsulated under these conditions, and direct exposure of C_60_ to liquid water or water vapor primarily resulted in exohedral oxidation, hydroxylation, or degradation of the carbon cage rather than the formation of H_2_O@C_60_ [6,34,36]. Consequently, the principal importance of HPHT methods in the context of H_2_O@C_60_ is historical. They demonstrated that intact fullerene cages can accommodate encapsulated species while also highlighting the limitations of physical encapsulation methods for more complex molecular guests.

### 2.2. Ion Implantation, Plasma and Arc-Discharge Routes

A second major class of preparation methods exploits energetic ions or plasma species to insert atoms into pre-formed fullerenes or to trap guest species during arc-discharge formation of the carbon cage [6,50]. Compared with HPHT loading, these approaches expanded the range of accessible atomic and metallic endofullerenes. However, their applicability remained largely restricted to atomic or metallic guests and did not provide a viable route for the encapsulation of neutral molecules such as H_2_O.

#### 2.2.1. Ion Implantation into C_60_ Films

In ion implantation, a beam of energetic ions (e.g., N^+^ or Li^+^) is directed onto a solid C_60_ target. Depending on the implantation energy, a fraction of ions penetrates the fullerene cage and becomes trapped as an endohedral guest [63,64]. A landmark example is the preparation of N@C_60_, in which electron paramagnetic resonance (EPR) spectroscopy demonstrated that the encapsulated nitrogen atom retains its atomic-like ^4^S_3_/_2_ electronic state inside the fullerene cage [63]. Subsequent developments enabled the preparation of additional atomic endofullerenes, including N@C_70_ and P@C_60_, confirming the broad applicability of ion implantation for selected atomic guests [63,65,66]. A common limitation of ion implantation is the trade-off between encapsulation efficiency and cage damage: increasing ion energy enhances the probability of cage penetration but simultaneously increases fullerene fragmentation and lattice damage, thereby limiting encapsulation efficiency [15,64,65].

#### 2.2.2. Plasma and Arc-Discharge Methods

Energetic plasma conditions likewise enable the formation of endohedral species. RF nitrogen plasma treatment of fullerene films generates nitrogen radicals and ions that can become encapsulated within pre-existing fullerene cages. Huang, Ata, and co-workers demonstrated the formation of ^14^N@C_60_ under such conditions, with EPR spectroscopy confirming the presence of atom-like nitrogen inside the carbon cage [67,68].

For endohedral metallofullerenes, the dominant preparation method remains arc-discharge synthesis. During vaporization of graphite electrodes doped with metals or metal oxides, fullerene cages are formed under high-temperature plasma conditions while simultaneously trapping metal atoms or clusters, leading to the formation of numerous metallofullerenes, including La@C_82_, Sc_3_N@C_80_, and related clusterfullerenes [6,15]. The composition of the encapsulated products can be tuned by varying the metal dopant, buffer gas, and arc-discharge conditions, enabling access to a broad family of endohedral metallofullerenes with diverse electronic and magnetic properties [15,69].

Although highly effective for electropositive metals and atomic guests, these methods are fundamentally unsuitable for the preparation of H_2_O@C_60_. Water molecules cannot survive the extreme temperatures of the arc plasma and, unlike atomic or metallic species, lack an efficient mechanism for selective encapsulation during fullerene cage formation. Consequently, neither ion implantation nor plasma- or arc-discharge methods have provided practical access to molecular water endofullerenes [6,34,36]. These limitations demonstrated that fundamentally different synthetic strategies were required for the encapsulation of neutral molecules. The solution to this challenge emerged with the development of molecular-surgery methodologies, discussed in the following section.

### 2.3. Chemical Routes and Post-Synthetic Encapsulation (“Molecular Surgery”)

#### 2.3.1. Development of the Molecular Surgery Concept

The controlled encapsulation of neutral molecular guests required a fundamentally different synthetic strategy, as conventional physical methods were ineffective for most small molecules [15,31,34,65]. Rubin and co-workers proposed that fullerene cages could be chemically opened, loaded with a selected guest molecule, and subsequently reclosed to regenerate the intact carbon framework. This approach, later termed “molecular surgery”, replaced physical encapsulation by a sequence of well-controlled organic transformations [54].

The practical development relied on the preparation of stable open-cage fullerene derivatives. Selective cleavage and enlargement of the C_60_ framework generated an orifice that communicated directly with the internal cavity. The resulting intermediates allowed guest insertion followed by stepwise reconstruction of the carbon cage [23,34,70,71].

A major proof of concept was the synthesis of H_2_@C_60_, in which molecular hydrogen was introduced into an open-cage derivative under pressure before restoration of the pristine fullerene framework. This work demonstrated that a closed neutral molecular endofullerene could be prepared from pristine C_60_ through organic synthesis, and established the basis for the encapsulation of more complex molecular guests [23,57].

#### 2.3.2. Evolution of Synthetic Strategies Toward H_2_O@C_60_ and Related Water Endofullerenes

An important precursor to the synthesis of closed H_2_O@C_60_ was reported by Iwamatsu and Murata in 2004. An open-cage C_60_ derivative with a large orifice reversibly accommodated one molecule of H_2_O or D_2_O. Although the cage was not subsequently reclosed and water could be released upon dehydration, the study demonstrated experimentally that water could enter the fullerene interior through a chemically generated opening [72].

When comparing molecular-surgery routes, it is important to distinguish between several measures of synthetic efficiency. In the following discussion, encapsulation efficiency (or filling/occupation level) refers to the fraction of open-cage fullerene molecules containing H_2_O after the loading step. Cage-closure yield refers to the chemical yield of one or more reactions converting a water-loaded open-cage intermediate toward the reconstructed C_60_ framework, whereas overall synthetic yield refers to the isolated yield of H_2_O@C_60_ over the specified synthetic sequence. Because different studies report overall yields from different starting intermediates, the yield basis must be stated explicitly when comparing synthetic strategies.

In 2011, Kurotobi and Murata reported the first synthesis of closed H_2_O@C_60_. Their multistep molecular-surgery protocol employed an open-cage C_60_ derivative containing an enlarged orifice, through which a single water molecule was introduced under high pressure before the fullerene framework was restored by a sequence of cage-closing reactions. The method produced bulk quantities of H_2_O@C_60_ suitable for structural and physicochemical characterization [36].

Krachmalnicoff, Levitt, and Whitby subsequently developed a more efficient and scalable route to H_2_O@C_60_ and D_2_O@C_60_. The improved procedure introduced phosphine-mediated partial closure and a Diels–Alder/retro-Diels–Alder sequence for final cage reconstruction. These modifications avoided the highest pressures required in the original route and substantially improved the overall synthetic yield [44]. Hashikawa and co-workers subsequently redesigned the geometry of the open-cage orifice using computational modeling to maximize both water uptake and cage closure efficiency. The resulting synthetic route increased the overall yield of H_2_O@C_60_ by approximately two- to five-fold compared with earlier procedures and demonstrated the value of computationally guided orifice design in molecular surgery [73].

The scope of water-endofullerene chemistry subsequently expanded beyond pristine C_60_. Molecular-surgery methodologies enabled the preparation of water-containing azafullerenes, including H_2_O@C_59_N and H_2_O@C_59_N^+^, which enabled the influence of nitrogen substitution and cage charge on the behavior of the confined water molecule to be investigated. In particular, H_2_O@C_59_N^+^ exhibits a strong, specific interaction between the encapsulated water molecule and the positively charged azafullerene cage [74,75]. Molecular surgery has also been extended to other neutral guests, including HF, CH_4_, and related small molecules, demonstrating the broader versatility of this synthetic strategy [31,34]. Finally, molecular surgery has enabled the preparation of isotopically labeled water endofullerenes, including D_2_O@C_60_ and H_2_^17^O@C_60_, as well as functionalized fullerene derivatives suitable for NMR, infrared, terahertz, dielectric, and neutron-scattering studies [22,37,39,40,44,76,77,78]. The availability of these materials subsequently enabled detailed structural, spectroscopic, and dynamical investigations of confined water. The principal experimental studies on the preparation and characterization of H_2_O@C_60_ and related water-containing fullerene systems are summarized in Table 1 and Table 2.

## 3. Structural and Spectroscopic Characterization of H_2_O@C_60_

**Figure 3:**
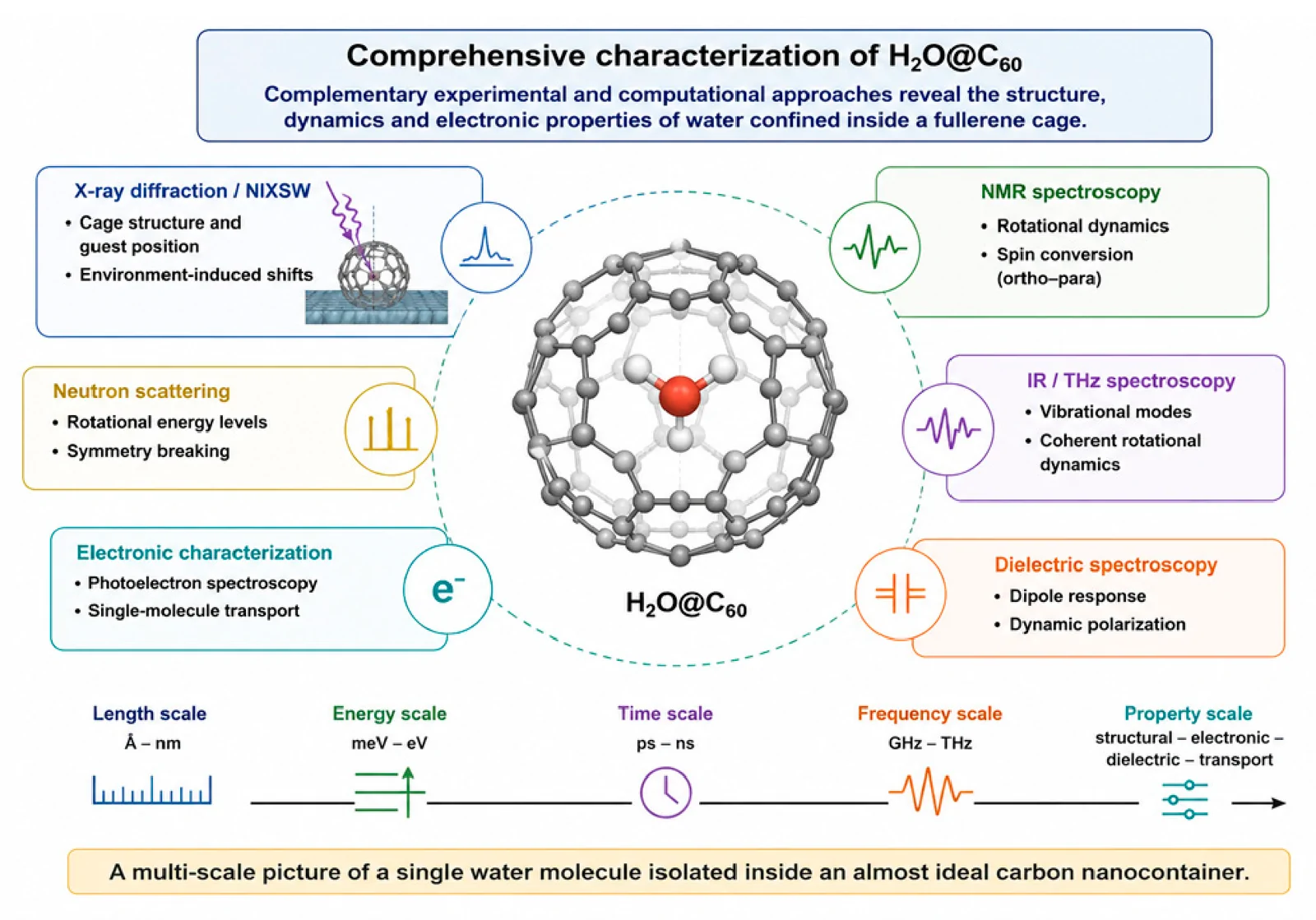
Complementary experimental methods for probing the structure, dynamics, and electronic properties of H_2_O@C_60_. Figure was created using OpenAI ChatGPT version 5.6 Sol.

The successful synthesis of H_2_O@C_60_ opened the way for comprehensive experimental investigations of the structural, spectroscopic, and physicochemical properties of a single water molecule confined within a fullerene cage. Because no single experimental technique can fully characterize such a nanoscale host–guest system, complementary approaches have been employed, including X-ray diffraction, nuclear magnetic resonance (NMR), vibrational spectroscopy, neutron scattering, dielectric measurements, and electronic characterization. Together, these complementary techniques provide a detailed experimental picture of confined water over a broad range of spatial and temporal scales (Figure 3) [36,37,39,40,42,76,77,78,80,83].

### 3.1. Structural Characterization

The first direct structural evidence for successful encapsulation of a water molecule inside the fullerene cage was provided by the single-crystal X-ray diffraction study accompanying the original synthesis of H_2_O@C_60_ reported by Kurotobi and Murata [36]. The crystallographic analysis demonstrated that incorporation of H_2_O produces essentially no measurable static distortion of the carbon framework: average C–C bond lengths remain nearly identical to those observed in pristine C_60_, indicating that the average cage structure remains close to the characteristic Ih geometry of C_60_ after guest encapsulation. This crystallographically averaged structural symmetry should, however, be distinguished from the effective local symmetry of the confining potential experienced by the dynamically moving H_2_O molecule. Electron-density maps further revealed that the oxygen atom is located close to the geometrical center of the fullerene cavity, providing the first direct experimental confirmation that the water molecule resides inside the carbon cage rather than interacting with its external surface [36]. Subsequent experimental studies confirmed that encapsulation of water has only a negligible influence on the geometry of the fullerene framework, demonstrating the remarkable rigidity of the C_60_ cage [36,42,83]. Complementary spectroscopic studies further showed that the encapsulated water molecule retains substantial translational and rotational mobility within the nearly spherical cavity [37,80,83]. Together, these studies show that the fullerene cage acts as a rigid nanoscopic container while allowing nearly unhindered motion of the confined water molecule [36,37,42,83].

Although conventional crystallographic measurements indicate that the average position of the oxygen atom coincides closely with the center of the fullerene cage [36], later surface-sensitive studies demonstrated that the location of the encapsulated water molecule may be influenced by the surrounding environment. Using normal-incidence X-ray standing wave (NIXSW) spectroscopy together with complementary photoelectron measurements, Jarvis and co-workers showed that adsorption of H_2_O@C_60_ on metallic substrates induces a slight displacement of the encapsulated molecule away from the cage center, reflecting subtle modifications of the internal potential by external electrostatic interactions. These perturbations affect the position and orientation of the confined water molecule, while leaving the fullerene framework essentially unchanged [89]. Overall, structural studies demonstrate that H_2_O@C_60_ combines a highly rigid carbon cage with a dynamically mobile guest molecule whose position can be subtly influenced by the surrounding environment [36,42,83,89].

These apparently different structural pictures are not mutually contradictory but rather reflect the different environments and timescales probed by the respective techniques. Conventional crystallography provides a spatially and temporally averaged description of crystalline H_2_O@C_60_ and therefore places the oxygen atom close to the center of an essentially undistorted C_60_ cage. By contrast, surface-sensitive experiments probe the endofullerene under a strongly asymmetric external environment, where adsorption-induced charge redistribution and the resulting intrafullerene electric field can shift the water molecule away from the cage center. Similarly, spectroscopic observations of rotational-level splitting, residual anisotropic interactions, or effective symmetry breaking do not necessarily require a large static deformation of the carbon framework. Even weak anisotropies in the local confining potential, arising from crystal packing, external fields, surface adsorption, or subtle cage polarization, may be sufficient to lift rotational degeneracies and preferentially bias the instantaneous position or orientation of the confined H_2_O molecule. Thus, H_2_O@C_60_ is best described as a system with a highly rigid average cage structure but a dynamically mobile guest whose local potential and effective symmetry remain sensitive to its surroundings.

### 3.2. Nuclear Magnetic Resonance Spectroscopy

Nuclear magnetic resonance (NMR) spectroscopy has played a central role in elucidating the local environment, molecular motion, nuclear-spin interactions, and spin-isomer conversion of water confined within C_60_. Unlike diffraction techniques, which provide an averaged structural picture, NMR provides information on the magnetic environment and dynamics of the encapsulated molecule. The suppression of intermolecular proton exchange and hydrogen bonding within the fullerene cage creates a well-defined system in which magnetic interactions that are normally obscured in bulk water can be resolved and quantified [22,36,38,77,80,91].

The initial characterization of H_2_O@C_60_ by Kurotobi and Murata included solution-state ^1^H and ^13^C NMR measurements that supported both successful water encapsulation and restoration of the closed C_60_ framework following molecular surgery [36]. The encapsulated water produced a distinct ^1^H resonance, whereas the ^13^C NMR spectrum retained the highly symmetric pattern characteristic of C_60_, indicating that encapsulation does not produce a large static perturbation of the carbon framework [36]. These initial observations established NMR as one of the principal techniques for investigating the structure and dynamics of confined water.

#### 3.2.1. Rotational Dynamics and Anisotropic Spin Interactions

**Figure 4:**
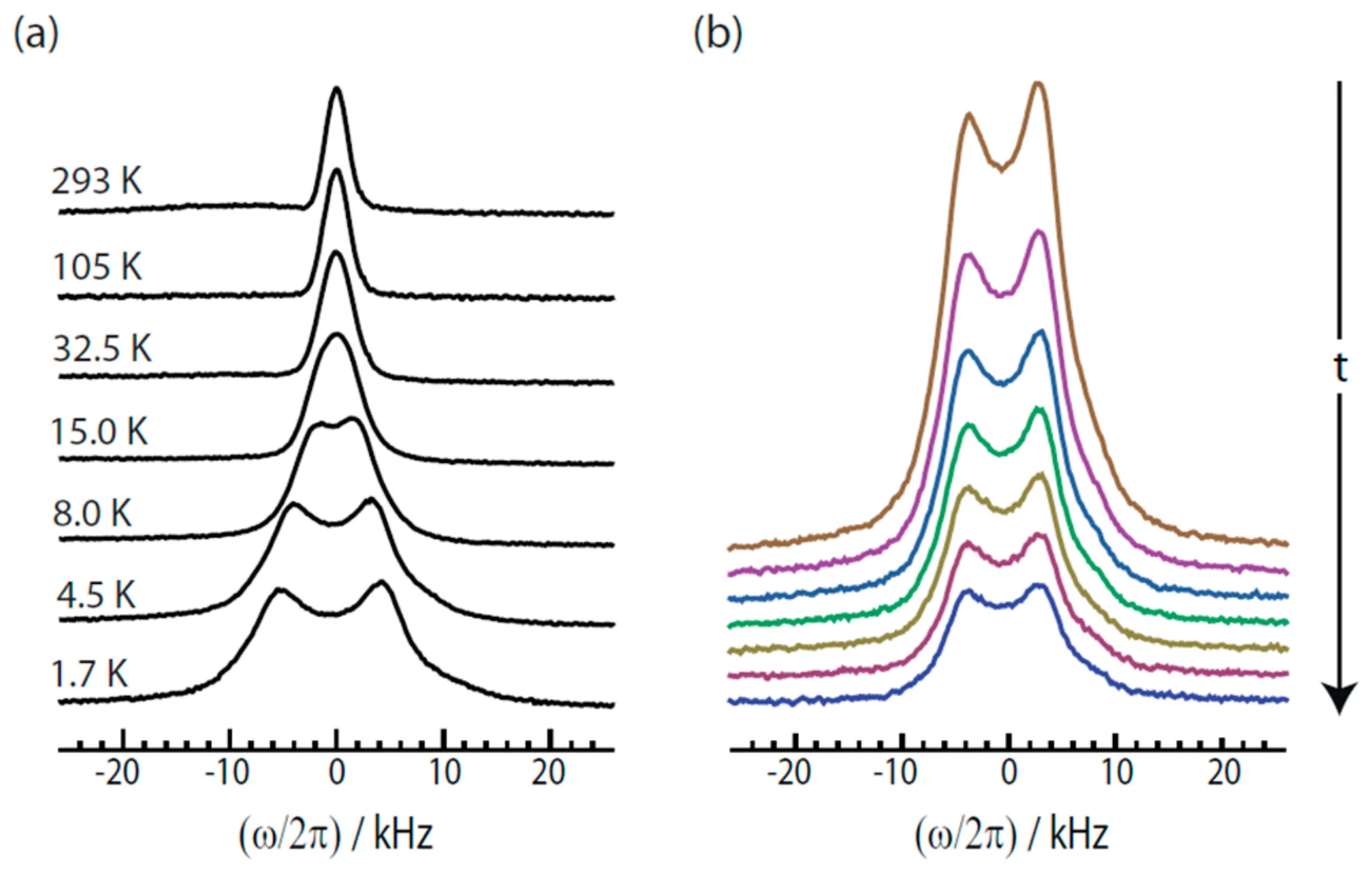
Temperature-dependent ^1^H NMR spectra of H_2_O@C_60_ showing the evolution of the proton resonance and ortho–para nuclear-spin conversion of the encapsulated water molecule. (**a**) ^1^H spectra from room temperature down to 1.7 K. (**b**) ^1^H spectra recorded at a set temperature of 5 K, taken at intervals of 2.25 h after cooling from 60 K. Reprinted with permission from [22].

Solid-state ^1^H NMR provided more detailed information on the rotational dynamics and anisotropic spin interactions of the confined water molecule. As illustrated in Figure 4, the proton resonance evolves markedly with decreasing temperature, reflecting changes in the rotational dynamics and spin interactions of the confined water molecule. Concistrè and co-workers combined static spectroscopy, magic-angle spinning, double-quantum-filtered dipolar recoupling, and numerical spin simulations to investigate anisotropic nuclear-spin interactions in H_2_O@C_60_ at room temperature [80]. They measured a residual ^1^H–^1^H dipolar coupling of approximately |b/(2π)| = 500 ± 10 Hz and a proton chemical-shift anisotropy (CSA) of approximately 3.1 ppm, with the principal axes of the dipolar and CSA tensors approximately perpendicular [80]. These residual interactions are indicative of rapid molecular motion that does not fully average the anisotropic magnetic interactions. The observed CSA could not be reproduced by a model based solely on partial orientational alignment of the water molecule. Concistrè and co-workers therefore proposed that slight geometrical distortion or electronic polarization of the fullerene cage contributes to the residual anisotropy [80].

#### 3.2.2. NMR of Isotopically Labeled Water Endofullerenes

**Figure 5:**
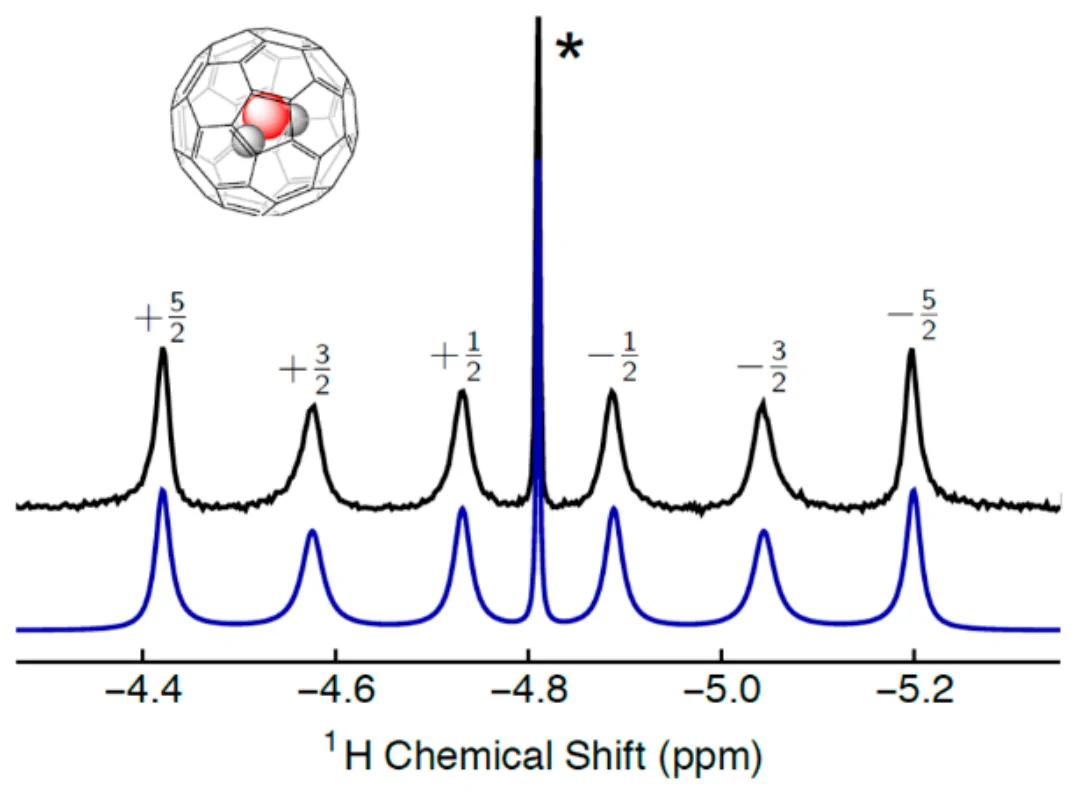
Experimental and simulated ^1^H NMR spectrum of H_2_^17^O@C_60_ showing the characteristic ^1^H–^17^O scalar-coupling sextet. The peak of the H_2_^16^O@C_60_ impurity is denoted by an asterisk. The inset shows the molecular structure. Reprinted with permission from [77].

The preparation of isotopically labeled water endofullerenes enabled multinuclear NMR studies of the confined molecule. Elliott and co-workers investigated H_2_^17^O@C_60_, in which the spin −5/2 ^17^O nucleus is scalar-coupled to the two equivalent water protons [77]. Because proton exchange between separate water molecules is suppressed by confinement within separate fullerene cages, the ^1^H NMR spectrum displayed a well-resolved sextet arising from ^1^H-^17^O scalar coupling. The six proton transitions exhibited a characteristic non-uniform linewidth pattern, which was explained by scalar relaxation of the second kind driven by quadrupolar relaxation of the ^17^O nucleus (Figure 5) [77]. Spectral analysis yielded a ^1^H–^17^O scalar coupling of approximately −77.9 Hz, a ^17^O quadrupolar coupling parameter of approximately 0.675 MHz, and a rotational correlation time of about 107 fs, consistent with sub-picosecond rotational diffusion of the encapsulated water molecule in solution [77].

The optimized synthesis of D_2_O@C_60_ also broadened the range of isotopically labeled water endofullerenes available for spectroscopic studies. Although this work was primarily synthetic, D_2_O@C_60_ provides a complementary isotopic system for studies of confined-water dynamics [44].

#### 3.2.3. Nuclear-Spin Isomerism and Spin Conversion

One of the most distinctive applications of NMR to H_2_O@C_60_ provides direct insight into the interconversion of the ortho and para nuclear-spin isomers of water. Ortho-H_2_O has a total proton nuclear spin of I = 1 and produces a ^1^H NMR signal, whereas para-H_2_O has I = 0 and does not contribute directly to the ^1^H NMR signal. Mamone and co-workers followed ortho–para conversion in solid H_2_O@C_60_ through the time-dependent intensity of the ^1^H NMR signal after temperature changes that altered the equilibrium spin-isomer populations [22]. At temperatures below approximately 15 K, the proton resonance broadened and developed a slightly asymmetric doublet, attributed mainly to proton–proton dipolar interactions associated with lifting of the degeneracy of the ortho-water rotational ground state. The ^1^H spin-lattice relaxation times remained below approximately 5 s throughout the investigated temperature range, and a T_1_ minimum near 8 K was linked to the low-energy splitting of the ortho-H_2_O ground rotational state [22].

Subsequent studies extended these investigations to room-temperature solution conditions. Meier and co-workers observed room-temperature spin-isomer conversion in H_2_O@C_60_ and reported quantum-rotor-induced nuclear polarization effects [91]. This result showed that the nuclear-spin dynamics of confined water are not restricted to cryogenic solids and that the coupling between rotational and nuclear-spin degrees of freedom remains experimentally accessible under chemically convenient conditions.

#### 3.2.4. Orientational Ordering in Anisotropic Media

NMR studies in anisotropic solvents provided an additional means of probing weak orientational effects. Kouřil and co-workers investigated H_2_O@C_60_, H_2_@C_60_, and HF@C_60_ dissolved in the nematic liquid crystal MBBA [38]. Partial ordering of the endofullerene molecules produced measurable residual dipolar couplings that would vanish in an isotropic solution. For H_2_O@C_60_, a proton dipolar splitting of approximately 670 Hz was observed at 300 K, corresponding to a small orientational order parameter [38]. The results were interpreted using a model in which a slightly anisotropic or distorted C_60_ cage aligns in the nematic medium and subsequently biases the orientation of the internal molecule [38]. These results show that the fullerene cage strongly, but not completely, isolates the guest from an anisotropic external environment.

Together, these NMR studies demonstrated the identity of the confined guest, characterized its rapid rotational motion and residual anisotropic interactions, and enabled ortho–para conversion to be followed in both solids and solutions [22,36,38,77,80,91]. NMR generally reports spatially and temporally averaged observables. Its interpretation therefore often requires numerical spin simulations or quantum-mechanical models, particularly when distinguishing between cage distortion, electronic polarization, and orientational effects.

### 3.3. Vibrational and Neutron Spectroscopies

The rotational and translational dynamics of the encapsulated water molecule have been investigated using infrared (IR), including far-infrared (FIR), terahertz (THz), and inelastic neutron scattering (INS) spectroscopies. These complementary techniques probe different aspects of the quantum dynamics of H_2_O confined within the fullerene cavity. Infrared spectroscopy primarily provides information on rovibrational transitions, terahertz spectroscopy probes low-energy rotational excitations and coherent rotational motion, whereas inelastic neutron scattering directly measures transitions between rotational quantum states. Together, these methods provide one of the most comprehensive experimental descriptions of an isolated water molecule available to date and consistently show that H_2_O confined inside C_60_ behaves as a nearly free quantum rotor, only weakly perturbed by the surrounding carbon cage [39,40,42,76,83].

**Figure 6:**
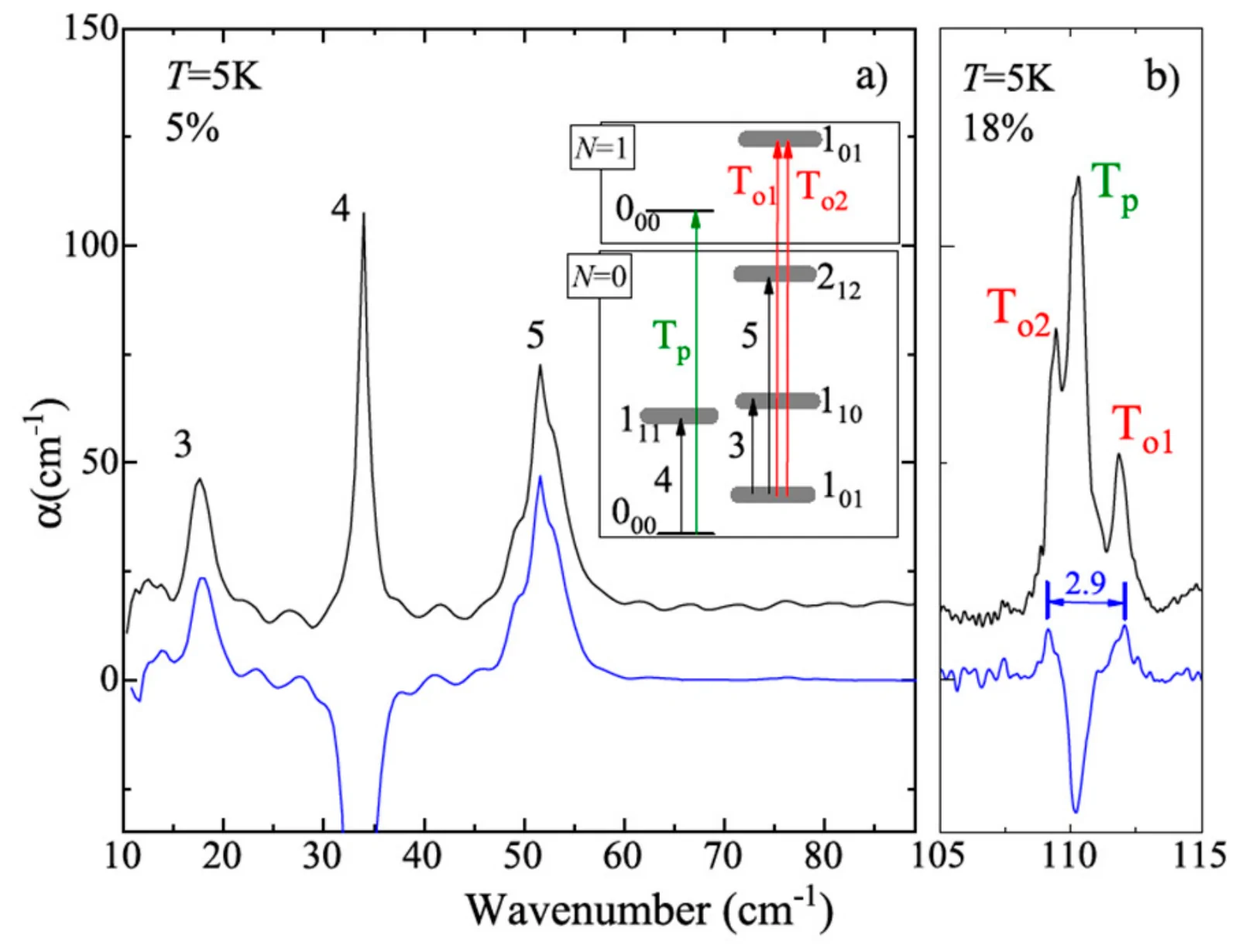
Far-infrared absorption spectrum of H_2_O@C_60_ showing the rotational transitions of ortho- and para-H_2_O together with the quantized translational mode. (**a**) Spectrum α(0) was measured after the temperature jump from 30 to 5 K (black), and the difference Δα = α(0) − α(Δt) was measured Δt = 44 h later (blue). The sample filling factor f = 0.05. Water rotational transitions corresponding to the absorption lines numbered 3 and 5 (ortho-water) and 4 (para-water) are shown in the inset. (**b**) Spectrum α(0) was measured after the temperature jump from 30 to 5 K (black), and the difference Δα = α(0) − α(Δt) was measured Δt = 5 h later (blue). The inset shows the corresponding rotational and translational energy-level scheme. Adapted from [76], licensed under CC BY 4.0.

Infrared spectroscopy has provided detailed information on the vibrational properties of encapsulated water. Early studies showed that the characteristic ν_1_, ν_2_, and ν_3_ vibrations remain readily identifiable after encapsulation, exhibiting only small red shifts relative to free water, consistent with weak host–guest interactions and preservation of the molecular structure [42,76]. A major advance was achieved by Shugai and co-workers, who assigned the principal rovibrational transitions of H_2_O@C_60_ using high-resolution cryogenic spectroscopy. Their work also revealed quantized translational motion of the confined molecule near 110 cm^−1^, crystal-field-induced splitting of rotational states, and an effective dipole moment of approximately 0.50 D, as illustrated in Figure 6. These findings demonstrate that confinement modifies the electrostatic environment inside the fullerene cage while preserving the molecular identity of H_2_O [76].

**Figure 7:**
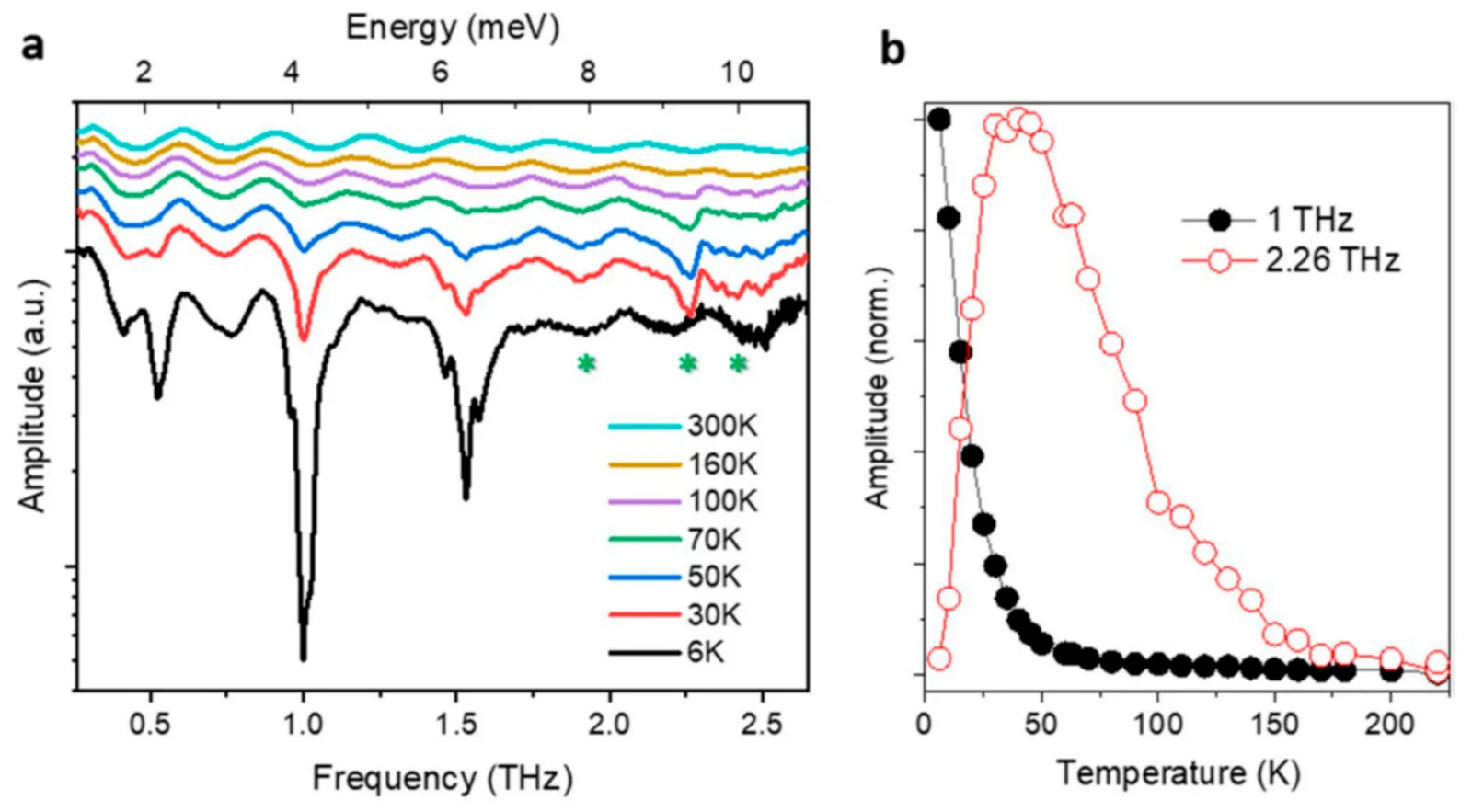
Temperature-dependent rotational spectra. (**a**), Selected Fourier spectra of the time-domain FID emissions of H2O@C60. The prominent peaks at 0.52 THz, 1 THz and 1.53 THz monotonically gain larger amplitude by lowering temperature and match well with rotational transitions of free water. The peak at 2.26 THz and two broad features at about 1.9 THz and 2.49 THz, marked by green asterisks, do not match the free water transitions (see text for details). The spectra are vertically displaced for clarity. Note the logarithmic scale of the vertical axis. Periodic oscillations are due to the Fabry–Pérot effect. (**b**), The peak amplitude of the transitions at 1 THz and 2.26 THz, normalized against their maximum values. Adapted from [40], licensed under CC BY 4.0.

Terahertz spectroscopy complements infrared measurements by probing low-energy rotational transitions that are inaccessible to conventional mid-infrared spectroscopy. Terahertz spectroscopy, together with far-infrared measurements, identified several low-energy rotational transitions over the temperature range 6–300 K, providing further experimental support for the rotational energy-level scheme established by neutron scattering [39,83]. More recently, time-domain terahertz spectroscopy enabled direct observation of coherent rotational dynamics in H_2_O@C_60_. Zhukov and co-workers employed single-cycle THz pulses to excite rotational motion of the encapsulated water molecule and monitored the subsequent coherent emission [40]. At temperatures below approximately 100 K, the rotational coherence persisted for more than 10 ps, indicating exceptionally weak environmental decoherence (Figure 7).

The observed rotational transitions closely resembled those expected for isolated gas-phase water, although additional spectral features near 2.26 THz suggested weak coupling between molecular rotation and lattice phonons of the fullerene crystal. Furthermore, time-resolved measurements directly monitored the slow ortho-to-para conversion of confined water following rapid cooling, providing an independent confirmation of the nuclear-spin conversion processes previously observed by NMR [22,40]. Together, infrared and terahertz spectroscopies demonstrate that confinement inside the fullerene cage only weakly perturbs the intrinsic rovibrational properties of water while simultaneously enabling observation of translational quantization, crystal-field effects, and exceptionally long-lived rotational coherence that would be difficult to study in condensed phases containing hydrogen-bonded water [39,40,76].

**Figure 8:**
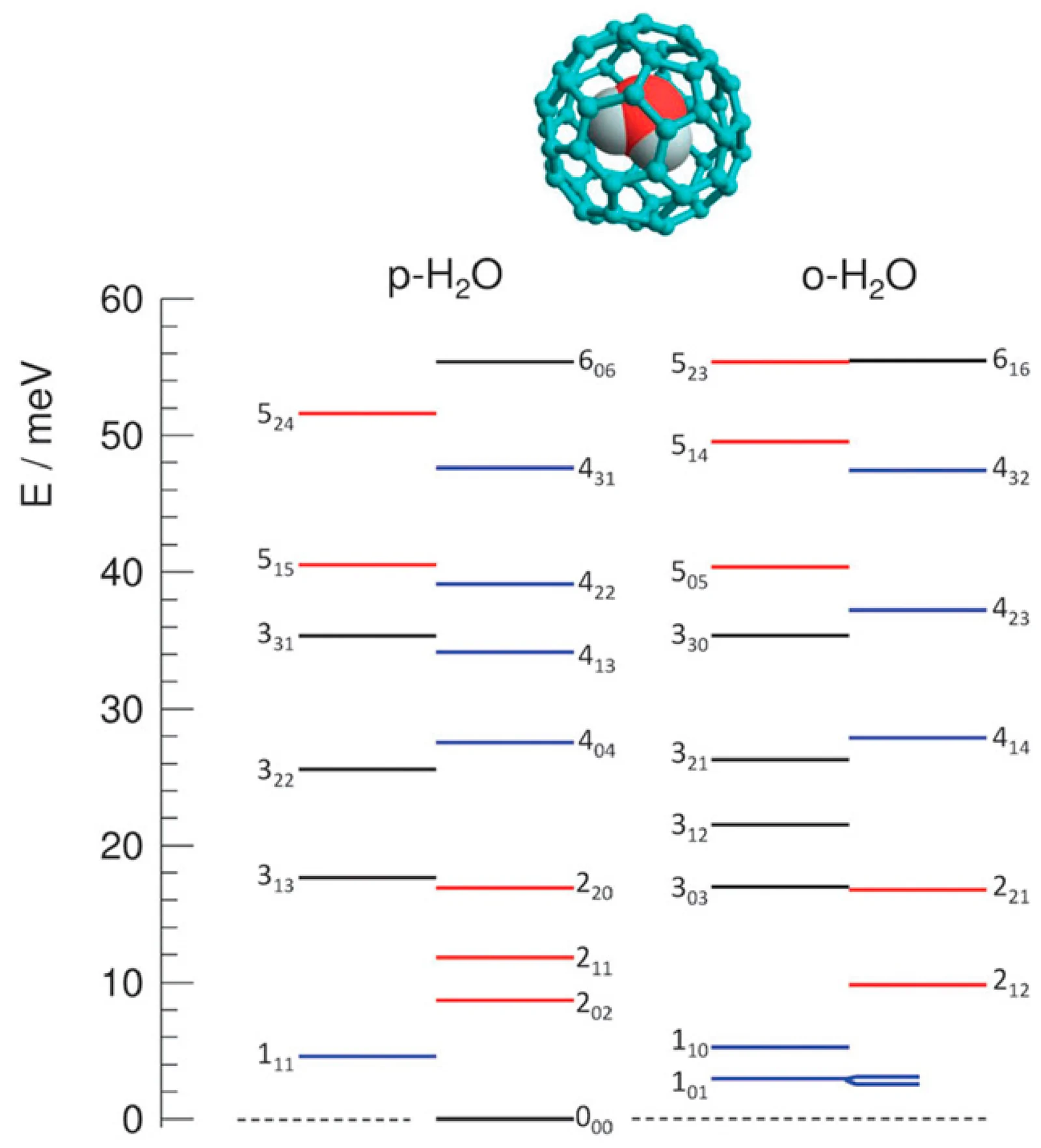
Rotational energy-level scheme of ortho- and para-H_2_O confined within C_60_ derived from inelastic neutron scattering measurements. Reprinted with permission from [83].

Whereas infrared and terahertz spectroscopies probe rovibrational excitations optically, inelastic neutron scattering provides complementary information by directly measuring transitions between rotational quantum states. Among all experimental techniques applied to H_2_O@C_60_, inelastic neutron scattering provides the most direct information on the rotational quantum states of the encapsulated molecule because neutron scattering directly probes transitions between rotational energy levels without relying on optical selection rules (Figure 8). The pioneering INS investigation by Goh and co-workers established the rotational energy-level scheme of H_2_O confined inside C_60_ and demonstrated excellent agreement with the spectrum expected for an almost freely rotating water molecule [83].

Both ortho- and para-H_2_O transitions were identified, while the relative intensities of the neutron peaks allowed direct monitoring of the slow nuclear-spin conversion process over many hours at cryogenic temperatures. Particularly significant was the observation that the triply degenerate rotational ground state of ortho-water is split into components with degeneracies of two and one, providing direct experimental evidence for a slight reduction in the effective symmetry experienced by the confined molecule. This splitting reflects weak anisotropies of the confining potential generated by the fullerene cage and the surrounding crystal environment rather than strong host–guest interactions [83]. Combined analysis of INS, infrared spectroscopy, NMR, and theoretical studies established a consistent picture of H_2_O@C_60_ as a nearly free quantum rotor undergoing quantized rotational and translational motion within the fullerene cavity [39,76,80,83].

Overall, vibrational and neutron spectroscopies consistently demonstrate that H_2_O@C_60_ occupies a unique position among molecular confinement systems. The fullerene cage isolates the water molecule from intermolecular hydrogen bonding while only weakly perturbing its intrinsic quantum states, thereby creating an almost ideal experimental realization of a freely rotating and translating single water molecule confined within a nanoscopic cavity [39,40,42,76,81,83].

### 3.4. Electronic and Dielectric Characterization

Although structural and spectroscopic techniques provide detailed information on the geometry and quantum dynamics of the encapsulated water molecule, they do not directly probe how its permanent electric dipole influences the electrical and electronic response of the host material. Dielectric measurements and electronic transport studies therefore provide an important complementary approach, allowing H_2_O@C_60_ to be studied under external electric fields over a broad range of frequencies and temperatures. Unlike ice or liquid water, where hydrogen-bonded networks dominate the dielectric response, H_2_O@C_60_ contains isolated molecular dipoles confined within separate carbon cages. It therefore provides a well-defined platform for investigating the dielectric behavior of isolated molecular dipoles without direct intermolecular hydrogen bonding [41,78,86,88,90].

#### 3.4.1. Dielectric Properties of H_2_O@C_60_ Crystals

The first dielectric investigation of crystalline H_2_O@C_60_ was reported by Aoyagi and co-workers, who combined single-crystal X-ray diffraction with temperature-dependent dielectric spectroscopy. The crystallographic analysis showed that the fullerene lattice remains essentially unchanged after encapsulation and that the water molecules are located close to the centers of the cages while retaining rotational mobility at low temperature. Dielectric measurements nevertheless revealed a measurable response to an external electric field despite the absence of direct hydrogen bonding between the confined molecules. The dielectric permittivity increased upon cooling and displayed frequency-dependent behavior, consistent with rotational polarization of the encapsulated molecular dipoles. No long-range ferroelectric ordering was detected down to 8 K. Instead, the results support a model in which water molecules behave as rotationally mobile dipoles with weak intermolecular coupling mediated through the fullerene lattice [41]. The observed response is therefore consistent with rotational polarization of screened molecular dipoles rather than the cooperative ordering characteristic of hydrogen-bonded ice [41].

#### 3.4.2. Electronic Effects of the Encapsulated Water Molecule

The permanent electric dipole of H_2_O has stimulated considerable interest in the electronic properties of fullerene-based materials and molecular devices. Although the fullerene cage efficiently screens the electric field generated by the encapsulated water molecule, both theoretical and experimental studies indicate measurable changes in molecular polarization, charge distribution, and electrostatic potential [86,89,90,92,93].

**Figure 9:**
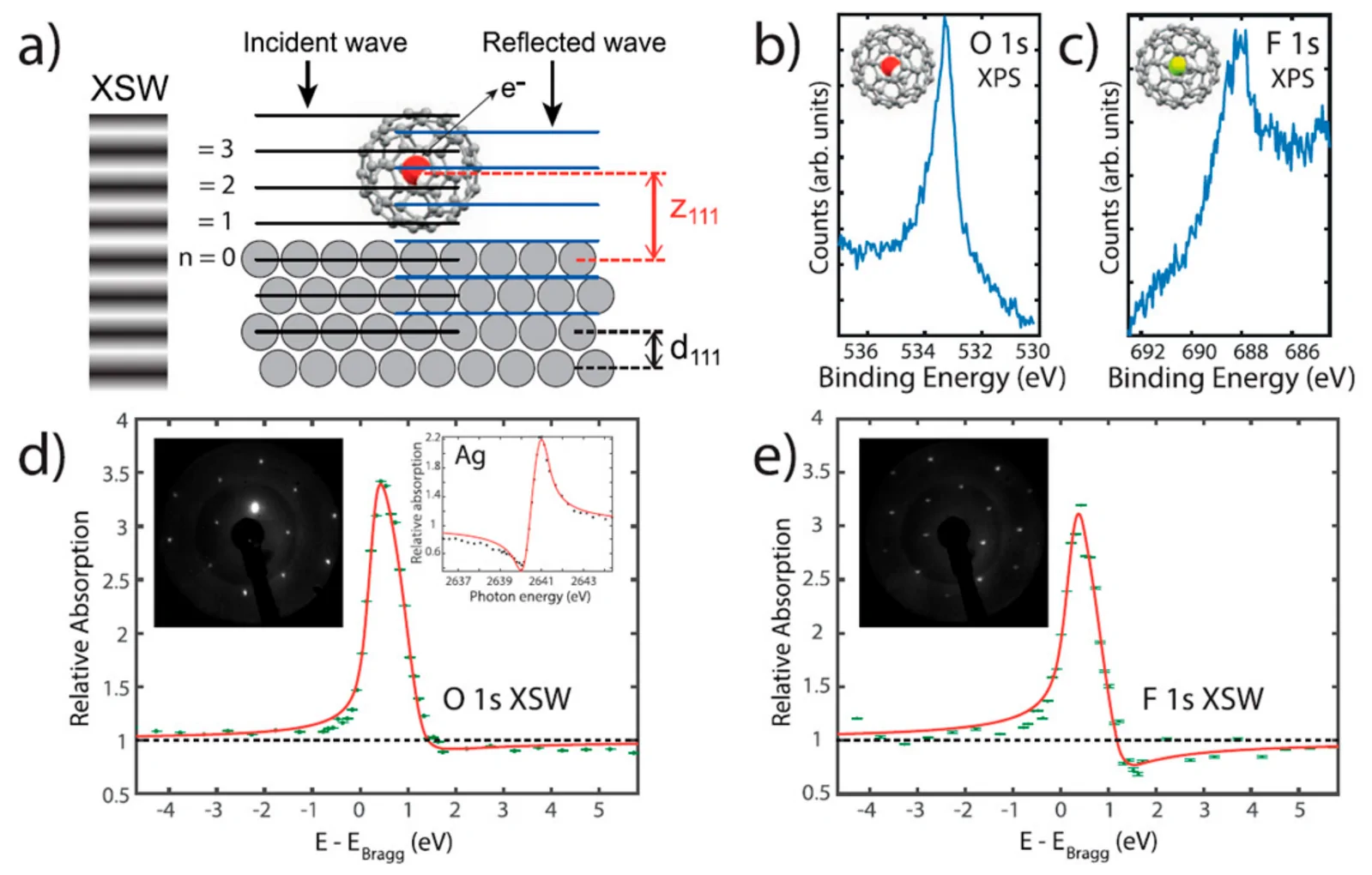
Locating trapped molecules with NIXSW. (**a**) Diagram of the NIXSW technique where an X-ray standing wave is established at the sample surface by tuning hν close to the crystal Bragg condition. (**b**) O 1s core-level photoemission spectrum (hν = 700eV) and (**c**) F 1s level (hν = 900 eV) for H_2_O@C_60_ and HF@C_60_, respectively, confirming the presence of the encapsulated molecules in the monolayer film. (**d**) NIXSW data for H_2_O@C_60_ with insets: bulk Ag NIXSW and low-energy electron diffraction (LEED, E = 46.5 eV). Fitting analysis results in a coherent position and coherent fraction of Pc = 0.35 ± 0.03 and Fc = 0.78 ± 0.05, respectively. (**e**) NIXSW data for HF@C60. Fitting analysis results in a coherent position and coherent fraction of Pc = 0.43 ± 0.03 and Fc = 0.72 ± 0.07, respectively. Inset, LEED (E = 50.5 eV). Adapted from [89], licensed under CC BY 4.0.

Surface-sensitive measurements have further demonstrated that the external environment can modify the electrostatic potential experienced by the confined water molecule. The experimental approach used to determine the position of the encapsulated water molecule within the fullerene cage is illustrated in Figure 9. Using normal-incidence X-ray standing wave (NIXSW) analysis supported by density functional theory and molecular dynamics simulations, Jarvis and co-workers determined that H_2_O occupies an off-center position within H_2_O@C_60_ adsorbed on Ag(111). This displacement was attributed to an inhomogeneous intrafullerene electric field generated by charge redistribution accompanying chemisorption of the C_60_ cage on the metal surface [89]. Complementary STM, ncAFM, and photoemission measurements revealed no discernible contribution of the encapsulated water molecule to the frontier orbitals and no measurable change in cage stiffness within the sensitivity of the applied methods. Thus, the fullerene shell provides substantial chemical and electronic shielding, but does not completely prevent an adsorption-induced electric field from influencing the position and dynamics of the encapsulated guest [89].

Single-molecule junction and transistor experiments have extended these observations by directly demonstrating electronic transport through H_2_O@C_60_, gate-dependent electrostatic effects, and inelastic transport features assigned to rotational excitations of the encapsulated water molecule [86,88,90]. These experimental advances have stimulated growing interest in H_2_O@C_60_ as a platform for molecular electronics and quantum-state readout. Its combination of chemical stability, a screened but finite electric dipole, quantized rotational states, and long-lived rotational coherence makes it an attractive model for investigating nanoscale dielectric response and electronically coupled molecular quantum dynamics [40,41,78,86,88,89,90]. Nevertheless, controlled manipulation of its orientation, nuclear-spin state, or rotational quantum state by an externally applied electric field remains at an early stage.

Overall, dielectric and electronic studies demonstrate that H_2_O@C_60_ combines efficient electrostatic screening with experimentally detectable dielectric and transport properties. Together with its long rotational coherence and nuclear-spin isomerism, these features make H_2_O@C_60_ a valuable model system for investigating confined molecular dipoles. The experimental findings summarized above also provide important benchmarks for computational studies of electrostatic screening, host–guest polarization, symmetry breaking, and translation–rotation coupling, which are discussed in the following section. Representative experimental studies discussed throughout this section are summarized in Table 2. Several of these investigations were complemented by quantum-chemical calculations, which are summarized separately in Table 3 and discussed in detail in Section 4.

## 4. Theoretical and Computational Studies

**Figure 10:**
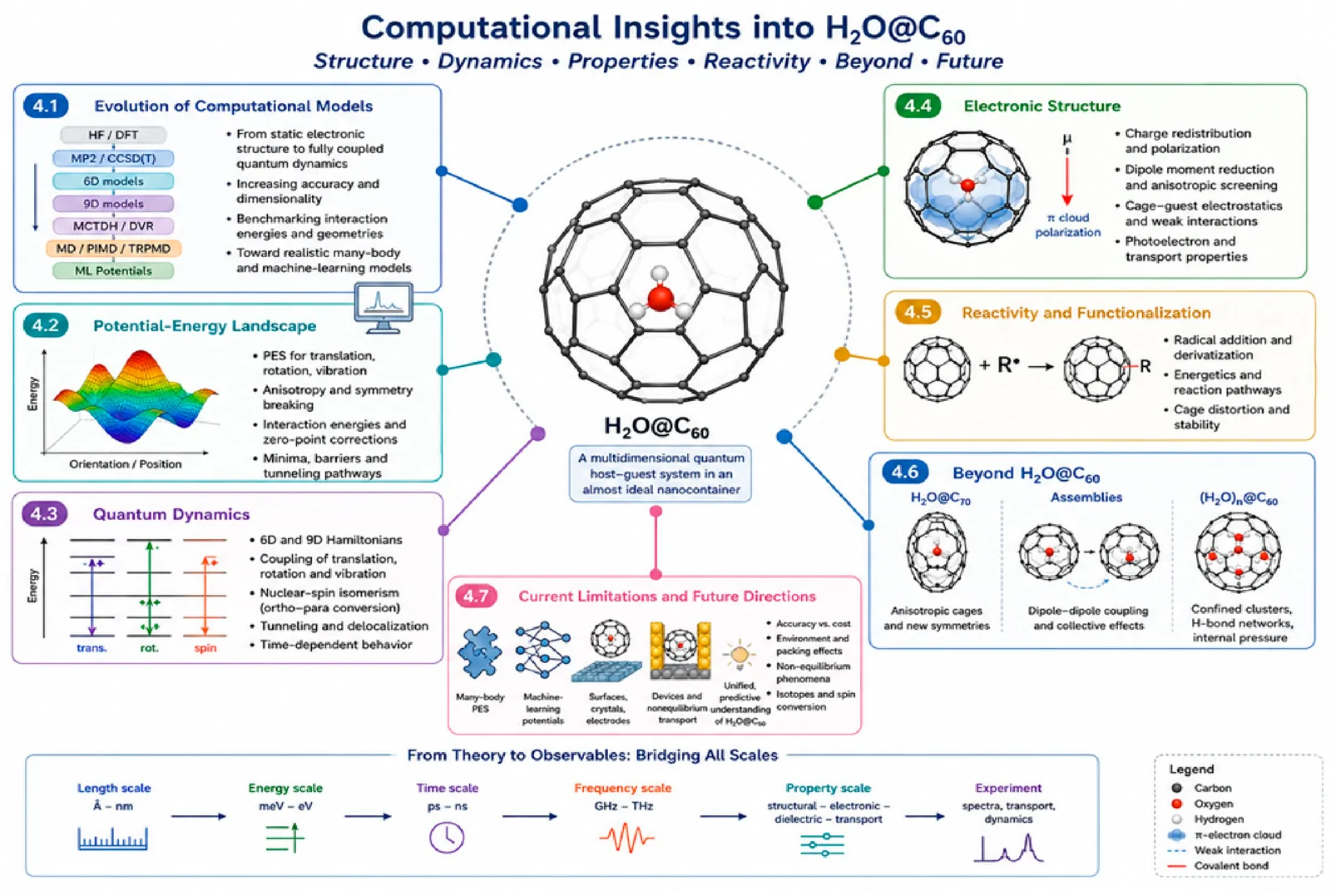
Conceptual roadmap of the computational approaches discussed in Section 4. This figure was created using OpenAI ChatGPT version 5.6 Sol.

Theoretical investigations have transformed H_2_O@C_60_ from an unusual endofullerene into one of the best-characterized model systems for molecular confinement at the nanoscale. Over the past two decades, computational approaches have evolved from static electronic-structure calculations toward fully coupled quantum-dynamical simulations capable of describing translation, rotation, vibration, electronic polarization, and environmental effects within a unified framework. The organization of this chapter follows this progression, beginning with the development of computational methods and continuing through interaction potentials, quantum dynamics, electronic properties, chemical reactivity, and emerging directions in endofullerene modeling, as summarized in Figure 10.

Computational studies have been indispensable for understanding H_2_O@C_60_ because many of its defining properties arise from coupled nuclear motions that cannot be fully resolved from experimental spectra alone. The fullerene cage provides a spatially well-defined but weakly anisotropic confinement potential, whereas the low mass, permanent dipole moment, nuclear-spin isomerism, and internal flexibility of H_2_O make the guest molecule sensitive to small variations in the host–guest interaction. Consequently, a physically meaningful description must account not only for equilibrium geometry and electronic structure but also for the quantization and mutual coupling of translational, rotational, and vibrational motions, as well as perturbations introduced by crystalline, surface, or device environment [93,94,95,96,97,98,99,100,101,102].

The theoretical treatment of water endofullerenes has evolved from static Hartree–Fock and density functional theory (DFT) calculations to correlated wave-function methods, symmetry-adapted interaction potentials, fully coupled six- and nine-dimensional quantum calculations, multiconfiguration time-dependent Hartree (MCTDH) approaches, molecular dynamics (MD), path-integral simulations, and machine-learning-assisted atomistic models. Together, these approaches have replaced simple static descriptions with a multidimensional picture in which H_2_O is dynamically delocalized, undergoes quantized translational and rotational motion, experiences weak but spectroscopically significant anisotropic interactions, and polarizes the π-electron system of C_60_ without forming a conventional covalent bond [93,94,95,96,97,98,99,100,101,102,103].

The studies discussed below include both calculations-only investigations and combined experimental–computational work. The former establish potential-energy surfaces, interaction mechanisms, and quantum-dynamical models, whereas the latter use calculations to interpret NMR, IR, INS, dielectric, electronic-transport, and surface-sensitive measurements. This distinction is useful because agreement with an experimental spectrum does not necessarily validate every component of the underlying theoretical model, while a high-level electronic-structure calculation may accurately describe an isolated complex but omit important crystal-packing, surface, electrode, or nonequilibrium effects [75,84,85,86,87,88,89,90,91].

### 4.1. Evolution of Computational Models for Water Endofullerenes

Early calculations on H_2_O@C_60_ addressed three fundamental questions: whether encapsulation is energetically favorable, whether the water molecule remains chemically and geometrically intact, and whether its permanent dipole significantly perturbs the electronic structure of the fullerene. Most static calculations predicted only small changes in the geometry of both H_2_O and C_60_ and identified the interaction as predominantly noncovalent, arising from dispersion, electrostatics, induction, and short-range exchange repulsion rather than through a conventional chemical bond [92,93,96,97].

These early models were necessarily limited because a single optimized structure represents only one minimum on a multidimensional potential-energy surface. In reality, the nearly spherical cavity of C_60_ permits numerous water positions and orientations of very similar energy. Using DF-LMP2 calculations benchmarked against DLPNO-CCSD(T), Dolgonos identified two low-energy structures of nominal C_1_ and C_2_ symmetry separated by only approximately 0.002 kcal mol^−1^, although only the C_1_ structure was a true minimum. The interaction energy was calculated as approximately −14.26 kcal mol^−1^ and changed only slightly, to −14.11 kcal mol^−1^, after inclusion of the zero-point-energy correction [96]. Such extremely small differences between orientational structures show why assigning a single rigid equilibrium orientation to confined H_2_O can be physically misleading.

**Figure 11:**
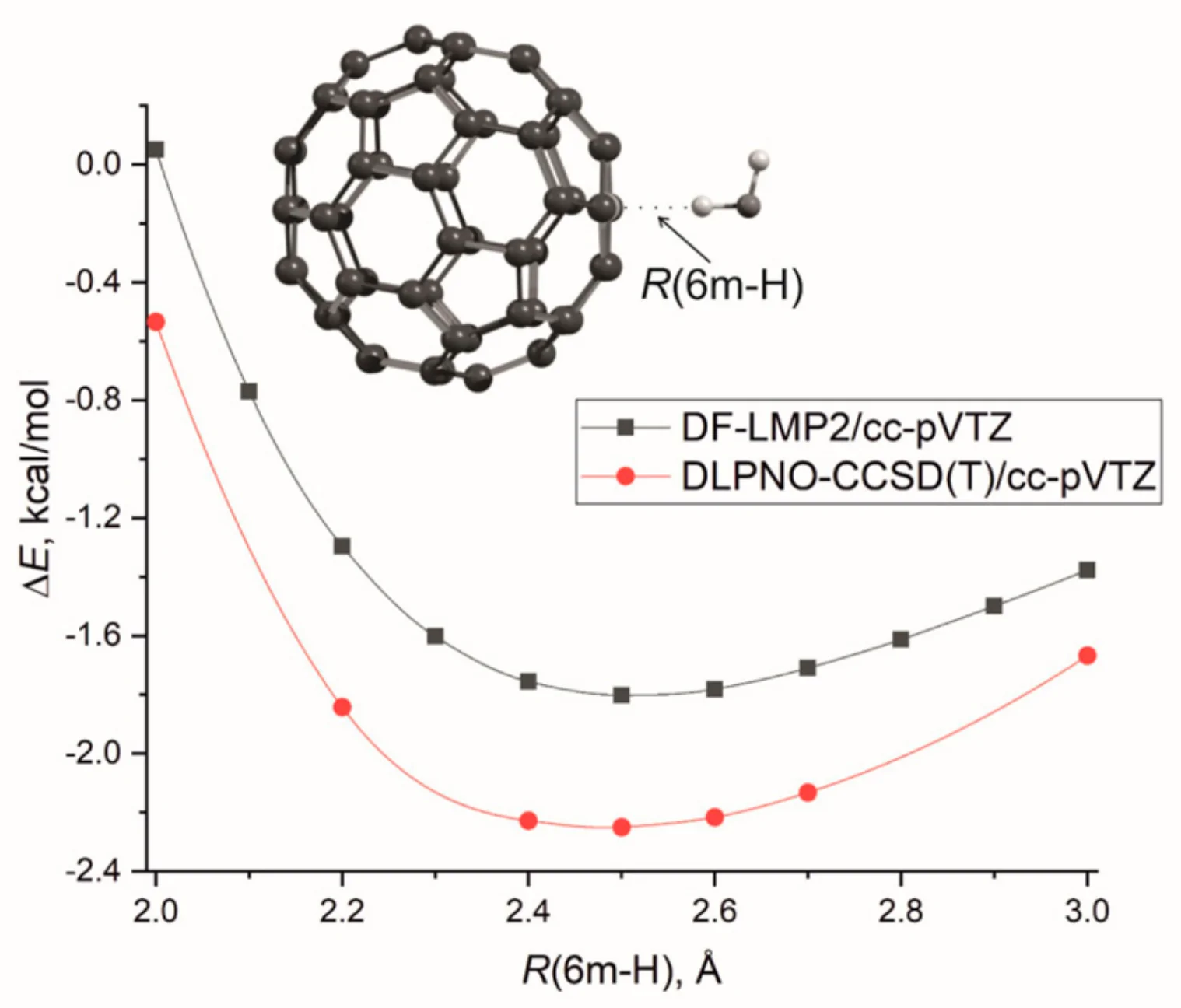
Comparison of the interaction-energy curves for the H_2_O–C_60_ system calculated using the DF-LMP2 and DLPNO-CCSD(T) methods. Reprinted with permission from [96].

The reliability of calculated interaction energies depends strongly on the electronic-structure method. Hartree–Fock calculations omit dispersion, while conventional semilocal DFT may describe it inadequately unless an explicit dispersion correction or a suitably parameterized functional is used. Because the attractive interaction is distributed over the entire curved π surface of C_60_, even relatively small errors in individual water–carbon contributions may accumulate. Correlated methods such as MP2 and coupled-cluster calculations, dispersion-corrected DFT, and DFT-SAPT-derived interaction potentials have therefore become particularly important for constructing physically realistic descriptions of the H_2_O–C_60_ potential, as illustrated by representative interaction-energy profiles shown in Figure 11 [93,95,96,97,100].

A further methodological transition involved moving from static electronic-structure calculations to explicitly dynamical models. Classical and ab initio MD provided information on finite-temperature rotation, structural fluctuations, and dipole-moment distributions. For example, Farimani and co-workers predicted rapid rotational motion of H_2_O inside C_60_ and showed that the energetic differences among alternative orientations are very small [104]. Nevertheless, classical MD cannot reproduce zero-point delocalization, rotational-level quantization, or ortho–para nuclear-spin restrictions, which become particularly important under cryogenic experimental conditions [92,103].

Rigorous quantum models therefore introduced six-dimensional Hamiltonians that included three center-of-mass translational and three rotational degrees of freedom for a rigid H_2_O molecule inside a rigid C_60_ cage. These were subsequently extended to nine dimensions by incorporating the two O–H stretching coordinates and the H–O–H bending coordinate. Twelve-dimensional and generalized multidimensional models were additionally developed to investigate interactions between the water molecules in neighboring H_2_O@C_60_ units [93,94,98,100,102].

The increasing dimensionality of these models is physically important rather than merely technical. A three-dimensional model describes only the translational “rattling” of the water center of mass. A six-dimensional treatment reveals translation–rotation coupling, while a nine-dimensional calculation permits intramolecular vibrations to respond to translation, rotation, and the surrounding cage. Models of interacting endofullerenes additionally address collective dipolar behavior and the extent to which a crystal of nominally isolated water molecules may develop intermolecular quantum correlations [93,94,98,100,102].

### 4.2. Geometry, Interaction Energies, and the Potential-Energy Landscape

Most electronic-structure calculations place the oxygen atom close to the geometrical center of C_60_, in broad agreement with the average position obtained crystallographically. However, “close to the center” does not imply that the molecule occupies a sharply localized and static position. The predicted equilibrium displacement depends on the electronic-structure method, the treatment of dispersion, whether C_60_ is considered rigid or flexible, and whether the system is modeled as an isolated molecule, crystal, adsorbed layer, or molecular junction. Because the radial confinement potential is shallow near its minimum, small off-center displacements may require little energy and may be further broadened by zero-point motion [94,95,96,97].

High-level calculations generally predict only minor deformation of the guest and host structures. In the DF-LMP2 study of Dolgonos, the O–H bonds were elongated by approximately 0.0082 Å and the H–O–H angle increased by about 0.9° relative to free H_2_O. The calculations also predicted frustrated translational modes at approximately 90.5–94.3 cm^−1^ and red shifts of about 19.2, 110.4, and 123.8 cm^−1^ for the bending, symmetric-stretching, and antisymmetric-stretching vibrations, respectively. These results agree qualitatively with the experimental conclusion that confinement preserves the chemical identity of water while measurably modifying both its inter- and intramolecular dynamics [96].

The term “hydrophobic confinement” is frequently used to describe H_2_O@C_60_, but it should not be interpreted as an absence of interaction. Pristine C_60_ does not contain an electronegative atom capable of forming a conventional O–H···X hydrogen bond with the guest. Nevertheless, the total attraction can be appreciable because the polarizable π-electron surface surrounds H_2_O on all sides. Dispersion accumulates over numerous water–carbon contacts, while permanent multipoles of water induce polarization of the electronic response in the cage. These attractive components are balanced at short range by Pauli repulsion. Topological analyses of the electron density have identified weak O···C and H···C contacts, but these are characterized predominantly as closed-shell interactions rather than conventional covalent or strong hydrogen bonds. Thus, for pristine H_2_O@C_60_, expressions such as “hydrogen bonding to the cage” should be used cautiously. A more appropriate description is that the host–guest interaction arises from the combined effects of dispersion, induction, electrostatic multipole interactions, and exchange repulsion within a highly polarizable nanoscale cavity [84,93,96,97].

Rashed and Dunn demonstrated that even a nominally high-symmetry host generates a nontrivial anisotropic potential for the guest. Their DFT-informed, symmetry-adapted treatment showed that a relatively small number of potential parameters can reproduce the essential features of the translation–rotation energy-level structure. This finding is particularly important because the experimentally observed splitting of the ortho-H_2_O ground rotational state depends less on the depth of the isotropic radial potential than on much smaller anisotropic contributions [97].

Although pristine H_2_O@C_60_ represents a nearly isotropic confinement environment, the potential-energy landscape changes more substantially when the carbon cage is chemically modified. In H_2_O@C_59_N, replacement of one carbon atom by nitrogen breaks the geometric and electronic equivalence of the cage atoms and creates a locally polar site. Hashikawa and co-workers considered several possible guest–host arrangements and, on the basis of variable-temperature NMR, relaxation measurements, and DFT calculations, proposed an attractive electrostatic interaction between the nitrogen-containing cage and the oxygen end of the confined H_2_O molecule. In the corresponding cation, H_2_O@C_59_N^+^, this interaction becomes stronger, leading to greater stabilization and more restricted water dynamics. Pristine H_2_O@C_60_ and H_2_O@C_59_N/H_2_O@C_59_N^+^ therefore represent two distinct confinement regimes. The former is dominated by spatially distributed weak interactions and extensive dynamical averaging, whereas the nitrogen-doped systems contain a specific electrostatic perturbation capable of biasing the position and orientation of the guest. Together, these systems demonstrate that subtle modifications of the carbon cage provide a rational strategy for tuning the structure, dynamics, and electrostatic environment of confined water [75].

### 4.3. Quantum Translation–Rotation–Vibration Dynamics

#### 4.3.1. Six-Dimensional Translation–Rotation Models

The nuclear dynamics of H_2_O@C_60_ cannot be described accurately by treating translation and rotation as independent. Displacement of the molecular center of mass changes the orientational potential, while reorientation of the molecular dipole and quadrupole moments changes the forces acting on the center of mass. This coupling produces a manifold of coupled translation–rotation eigenstates rather than independent translational and free-rotor energy ladders [97,98].

Felker and Bačić reported rigorous six-dimensional calculations for the coupled translation–rotation eigenstates of para- and ortho-H_2_O@C_60_. Both the water molecule and the fullerene cage were treated as rigid, and the host was assumed to preserve ideal *I*h symmetry. The calculated states were classified using irreducible representations of the relevant molecular symmetry group and were assigned translational, rotational, orbital-angular-momentum, and total-angular-momentum quantum labels. The calculated level structure permitted tentative assignments of several INS transitions that could not be interpreted using a simple free-rotor model [98]. These calculations demonstrated that H_2_O inside C_60_ is neither a static inclusion nor a completely free gas-phase rotor. Translation and rotation are intrinsically coupled by confinement, even when the cage is perfectly rigid and icosahedral. The idealized *I*h model also provides a useful reference: additional splittings observed experimentally must arise from perturbations not included in the isolated, perfectly symmetric model, such as cage deformation, crystal packing, neighboring fullerenes, surfaces, or external electric fields [98,99].

#### 4.3.2. Origin of Symmetry Breaking

INS and low-temperature NMR experiments revealed that the nominally triply degenerate ground rotational state of ortho-H_2_O is split into a doublet and a singlet. Because ideal I_h symmetry does not generate this pattern, several computational mechanisms have been investigated to identify the physical origin of the effective symmetry reduction [95,97,98,99]. One proposed mechanism is electric dipole–dipole coupling between water molecules encapsulated in neighboring cages. Felker and Bačić examined this effect through twelve-dimensional calculations for a dimer of H_2_O@C_60_ units. Translation–rotation states of the two monomers were coupled through the water–water dipole interaction. The calculated splittings depended on the effective water dipole moment, the separation between the endofullerenes, and the nature of the underlying monomer states. The results showed that intermolecular dipolar coupling can influence the low-energy spectrum, although it does not necessarily provide a complete explanation for all observed splittings [100].

The same framework was generalized to linear assemblies of several H_2_O@C_60_ units. These calculations revealed two classes of collective states and showed that selected low-energy states may develop increasing dipolar order with increasing assembly size. The results are relevant to the interpretation of crystalline H_2_O@C_60_ because they demonstrate that quantum rotational delocalization and dipole coupling must be considered simultaneously; the system cannot be reduced to a classical lattice of rigid, statically aligned water dipoles [101]. A different explanation was advanced by Felker and co-workers, who modeled a central endofullerene surrounded by twelve nearest-neighbor C_60_ molecules in the orientationally ordered solid. Electrostatic interactions between the guest and the charge distributions of neighboring fullerene cages generated splittings in good agreement with experiment for the dominant crystallographic orientation. This work identified guest–neighbor multipolar interactions—especially quadrupolar components—as a plausible major contribution to the observed symmetry breaking [99].

**Figure 12:**
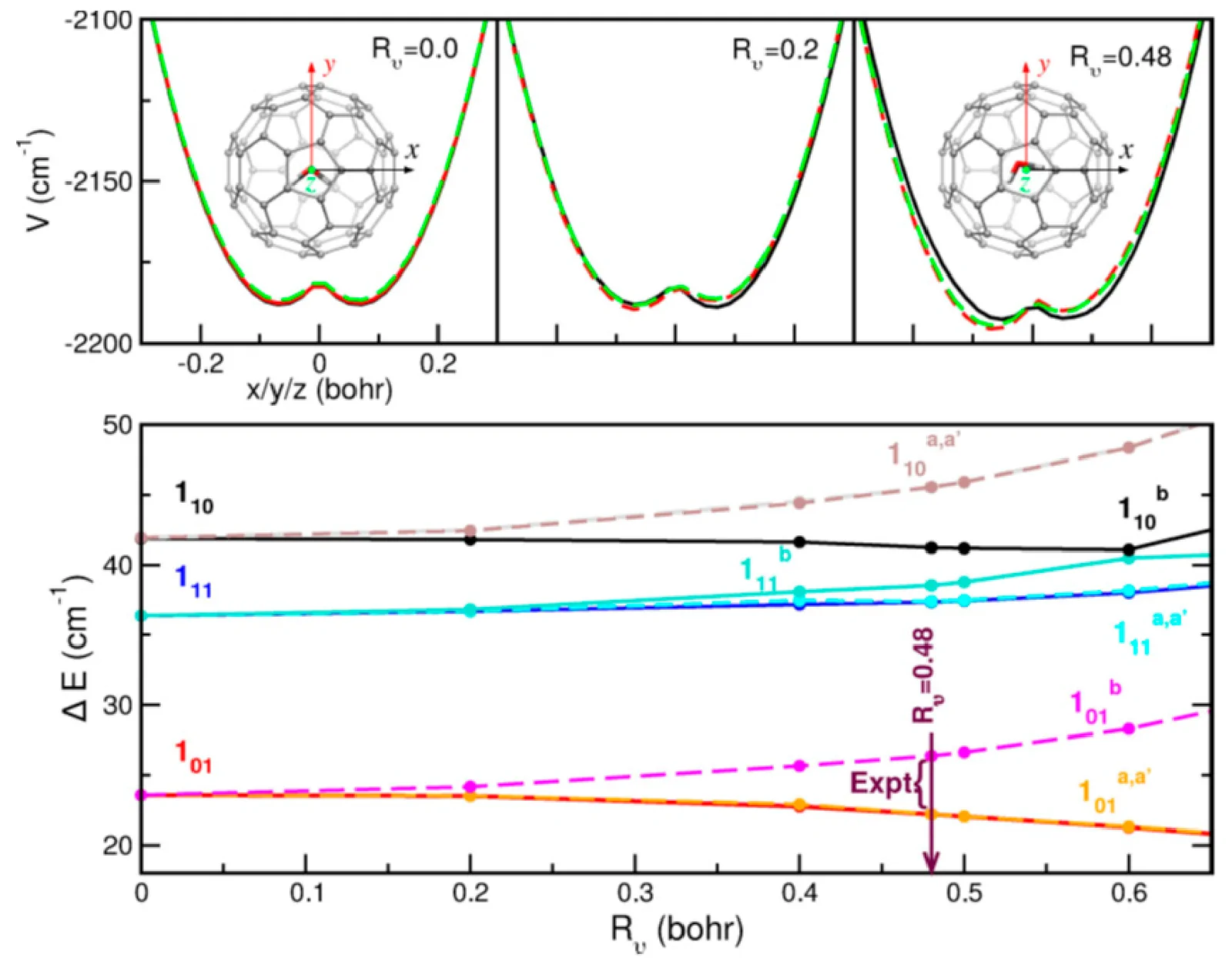
Effect of anisotropic confinement on the potential-energy landscape and rotational energy levels of H_2_O@C_60_. Adapted from [95] licensed under CC BY 3.0.

Carrillo-Bohórquez and co-workers subsequently examined two competing mechanisms using fully coupled MCTDH calculations: displacement of H_2_O away from the center of an otherwise ideal cage and uniaxial distortion of the C_60_ framework. Both models reproduced the qualitative singlet–doublet degeneracy pattern, but the uniaxial cage-deformation model provided better quantitative agreement with the experimental splitting. The effect of anisotropic confinement on the potential-energy landscape and the resulting splitting of the lowest rotational states is illustrated in Figure 12.

The authors therefore concluded that uniaxial cage distortion provided the most plausible explanation within the models considered, while emphasizing that a definitive conclusion requires more accurate many-body potentials including both the endohedral and exohedral environments [95]. These mechanisms should not be treated as mutually exclusive. Off-center displacement, cage deformation, neighboring-cage electrostatics, dipole–dipole coupling, and crystal packing are likely to act simultaneously. Their relative contributions can also vary between an isolated molecule, crystal, adsorbed monolayer, and single-molecule electronic device. The robust theoretical conclusion is therefore that the observed symmetry breaking originates from weak perturbations beyond the ideal isolated *I*h model, rather than from a strong directional chemical bond between water and pristine C_60_ [95,97,99,100,101].

#### 4.3.3. Fully Coupled Nine-Dimensional Treatments

Six-dimensional calculations treat H_2_O as a rigid body and therefore cannot describe changes in O–H distances, the H–O–H angle, or the water vibrational frequencies. Felker and Bačić extended their approach to a fully coupled nine-dimensional Hamiltonian including three translational coordinates, three rotational coordinates, and the three intramolecular vibrational coordinates of H_2_O. The Hamiltonian included translational kinetic energy, vibration–rotation and Coriolis interactions, intramolecular kinetic energy, and a multidimensional potential-energy surface. The nine-dimensional calculations showed that internal flexibility is necessary for a unified description of low-frequency translation–rotation states and high-frequency water fundamentals. The calculations further demonstrated that vibrational excitation modifies the translation–rotation spectrum and that averaging over large-amplitude intermolecular motion alters the confinement-induced frequency shifts. However, full convergence is computationally demanding because low-frequency delocalized motions must be represented simultaneously with localized O–H stretches [102].

Carrillo-Bohórquez, Valdés, and Prosmiti developed a complementary full-dimensional MCTDH approach based on an exact kinetic-energy operator in Radau coordinates and an n-mode representation of the potential-energy operator. All nine degrees of freedom of H_2_O were treated explicitly. The calculations reproduced confinement-induced splitting and vibrational shifts and showed that the accuracy of the nuclear-dynamics method is inseparable from the quality of the underlying water–cage potential [94].

The symmetry-adapted variational and MCTDH approaches offer complementary advantages. Symmetry-based calculations facilitate state classification and interpretation in terms of angular-momentum coupling, whereas MCTDH is well suited to high-dimensional, strongly coupled dynamics and flexible potential representations. Together, these methods support the view that H_2_O@C_60_ is a highly coupled nuclear quantum system despite the chemically weak nature of the guest–host interaction [94,98,102].

#### 4.3.4. Nuclear Quantum Effects and Finite-Temperature Dynamics

**Figure 13:**
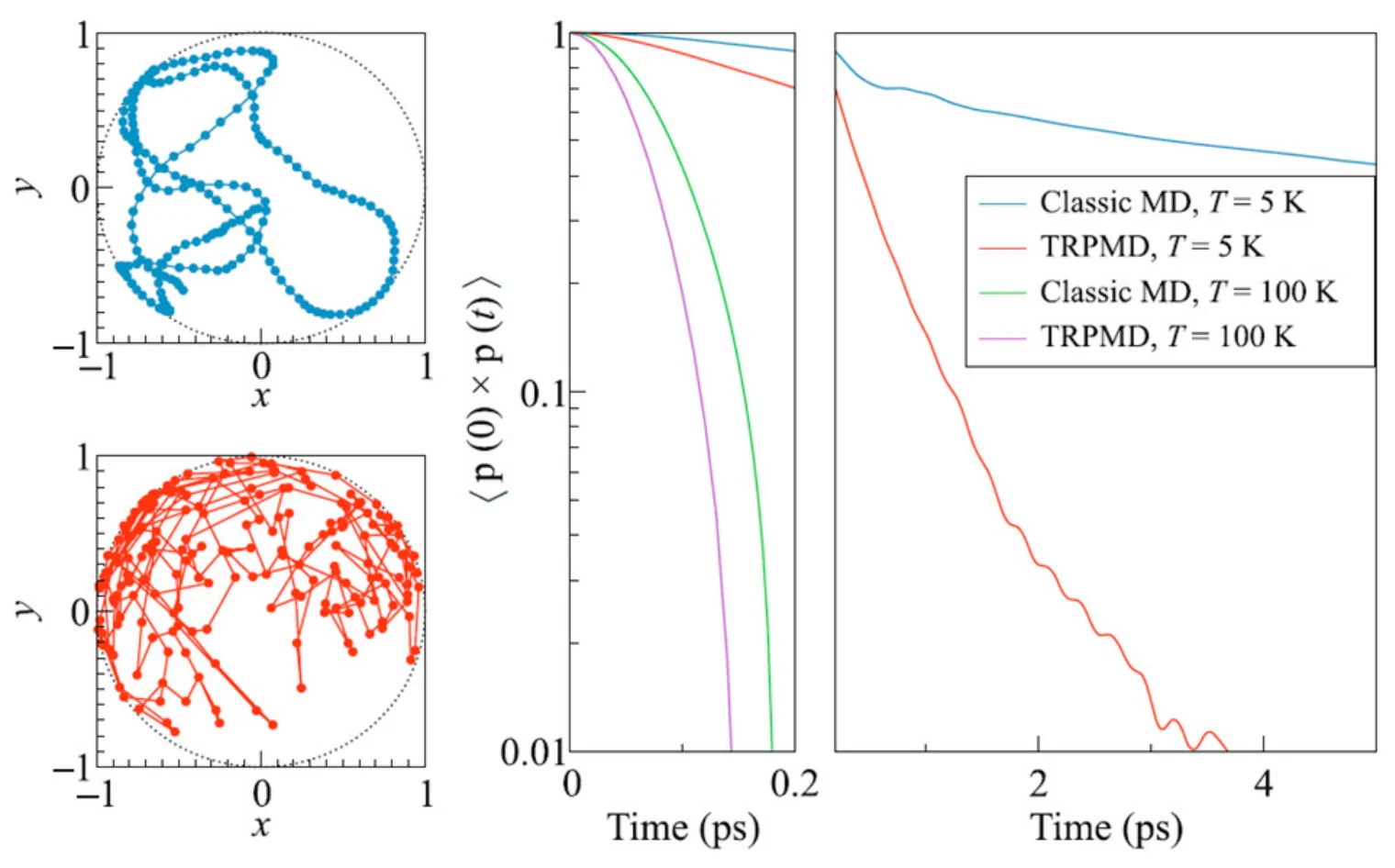
Comparison of classical molecular dynamics (MD) and thermostated ring-polymer molecular dynamics (TRPMD) simulations of H_2_O@C_60_. Reprinted with permission from [103].

Classical MD is useful for examining rotational diffusion and thermal fluctuations but becomes inadequate at cryogenic temperatures, where zero-point motion, nuclear delocalization, and discrete rotational states dominate. Potapov and Kondratyuk addressed this limitation by combining a machine-learning moment tensor potential trained on DFT trajectories and thermostated ring-polymer molecular dynamics at 5 K. Representative results illustrating the influence of nuclear quantum effects on the rotational dynamics of confined H_2_O are shown in Figure 13, where inclusion of nuclear quantum effects leads to enhanced orientational delocalization and faster rotational relaxation of the confined water molecule.

Inclusion of nuclear quantum effects broadened the bending and O–H stretching bands, increased the spatial delocalization of the hydrogen nuclei, and strongly changed the calculated rotational relaxation. The dipole autocorrelation decay time was approximately 1 ps in the quantum simulation, compared with approximately 27 ps in classical MD. The authors also reported a shortening of the mean O–H bond relative to bulk water and a broader quantum-mechanical distribution of bond lengths. Machine-learning potentials have emerged as an effective link between electronic-structure calculations and statistically meaningful finite-temperature simulations. Nevertheless, their transferability depends on the training set. A potential trained predominantly near equilibrium may not accurately describe rare off-center configurations, significant cage deformation, or high-energy regions sampled by quantum delocalization. Moreover, the moment tensor potential used by Potapov and Kondratyuk did not explicitly incorporate long-range interactions between neighboring endofullerenes, thereby restricting the model primarily to isolated H_2_O@C_60_ [103].

### 4.4. Electronic Structure, Polarization, and Electrostatic Shielding

#### 4.4.1. Reduction in the Effective Dipole Moment

**Figure 14:**
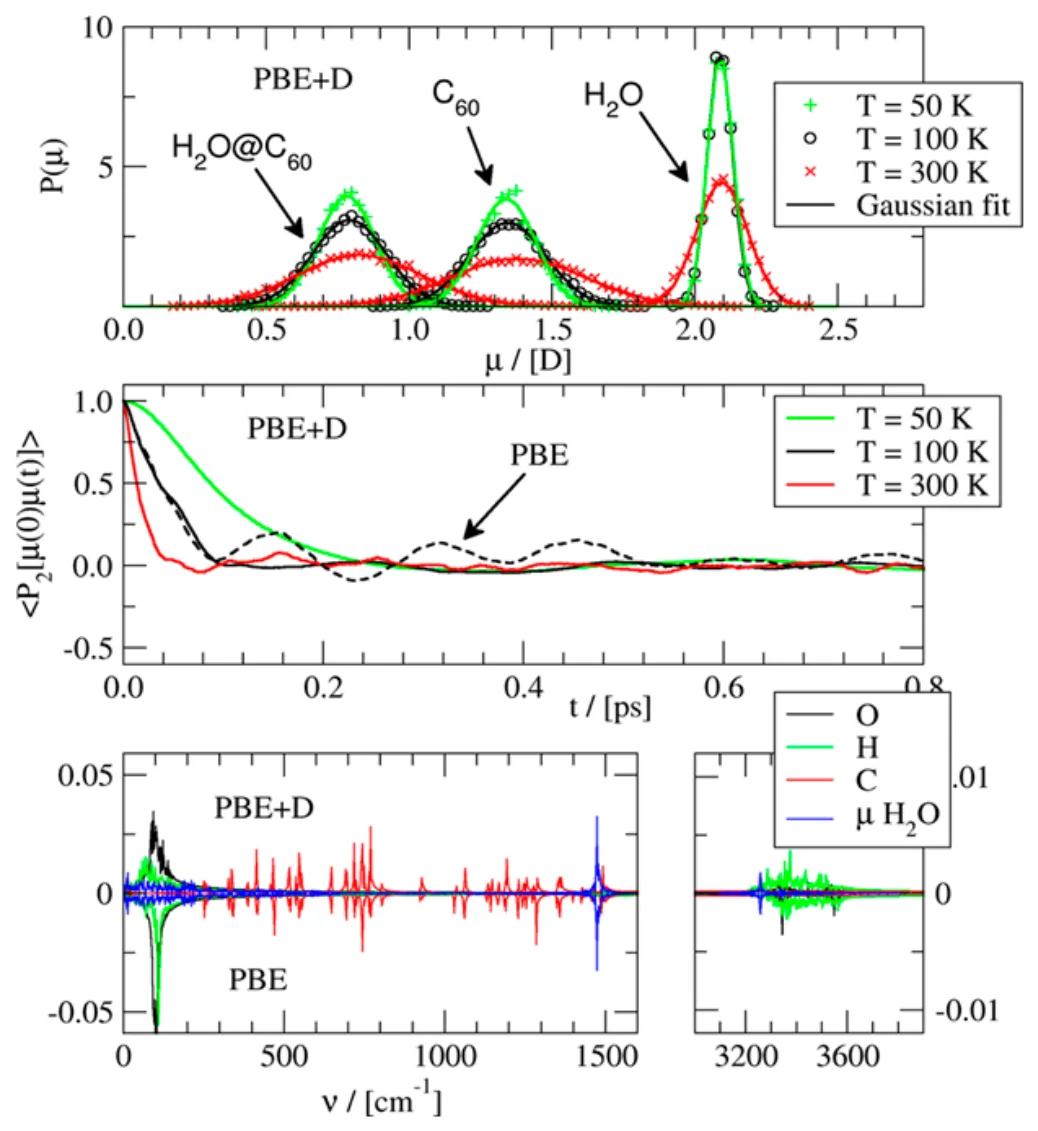
Ab initio molecular dynamics results for H_2_O@C_60_, including dipole-moment distributions (**top**), dipole orientational autocorrelation functions (**middle**), and calculated infrared spectra (**bottom**). Reprinted with permission from [93].

One of the most important electronic consequences of encapsulation is the reduction in the observable dipole moment of H_2_O@C_60_ relative to isolated water. This reduction does not mean that the internal H_2_O molecule becomes nonpolar. Instead, the delocalized π-electron cloud of C_60_ polarizes in the opposite direction and produces an induced counter-dipole that partially screens the guest dipole [84,93]. Ensing, Costanzo, and Silvestrelli separated the total dipole into guest and cage contributions using DFT, Wannier-center analysis, and ab initio MD. The water molecule retained a dipole moment of approximately 2.01 D, whereas the C_60_ cage acquired an induced dipole of approximately 1.46 D in the opposite direction, yielding a total dipole of about 0.55 D. Their analysis helped reconcile earlier discrepancies between calculations predicting either near-complete retention or strong reduction in the water dipole. As shown in Figure 14, polarization of the C_60_ cage substantially reduces the observable dipole moment of the endofullerene while preserving the intrinsic polarity of the confined H_2_O molecule [93].

Torii and co-workers refined this interpretation through temperature-dependent IR spectroscopy supported by DFT calculations. Their electron-density analysis indicated a single-sided but delocalized polarization of the fullerene π system. Electron density was redistributed primarily within and across the cage rather than transferred directly between H_2_O and C_60_. This response accounted for both the reduced effective dipole moment and the low IR intensities of the water vibrational bands [84].

The commonly used description of C_60_ as a molecular “Faraday cage” is therefore informative but incomplete. The carbon cage strongly screens the electric field generated by the guest when viewed externally, yet it is itself polarizable and does not provide absolute electrostatic isolation. Weak external fields, surfaces, or charged functional groups can modify the potential experienced by the internal molecule even when the frontier molecular orbitals remain almost entirely localized on C_60_ [84,86,93].

#### 4.4.2. Frontier Orbitals and Charge Redistribution

Most calculations predict that encapsulated neutral water has only a limited effect on the HOMO and LUMO energies of isolated C_60_. The frontier orbitals remain predominantly cage-centered, with negligible direct contribution from water orbitals. This conclusion agrees with single-molecule conductance measurements by Kaneko and co-workers, in which H_2_O@C_60_ and empty C_60_ exhibited comparable conductance. DFT calculations supported effective screening of the guest and the absence of direct electronic contact between water and the gold electrodes [86]. However, small changes in orbital energy do not imply an absence of electronic polarization. Liu and co-workers obtained similar HOMO and LUMO energies for isolated C_60_ and H_2_O@C_60_ but found a redistribution of the calculated frontier-orbital density. Experimentally, H_2_O@C_60_ single-molecule transistors displayed stronger gate coupling than comparable C_60_ devices. The authors attributed this difference tentatively to water-induced polarization and displacement of the molecular electron density, although junction geometry and dielectric effects could also contribute [90]. The results of Kaneko and Liu are not necessarily contradictory. Low-bias conductance through a two-terminal molecular junction may remain dominated by the C_60_ π system, whereas the electrostatic response of a three-terminal transistor may be sensitive to subtle changes in molecular polarization, capacitance, orientation, and coupling to the gate. Calculations intended to model device behavior must therefore include realistic electrodes and boundary conditions rather than extrapolating directly from isolated-molecule DFT [86,90].

#### 4.4.3. Electron Transport Coupled to Water Rotation

**Figure 15:**
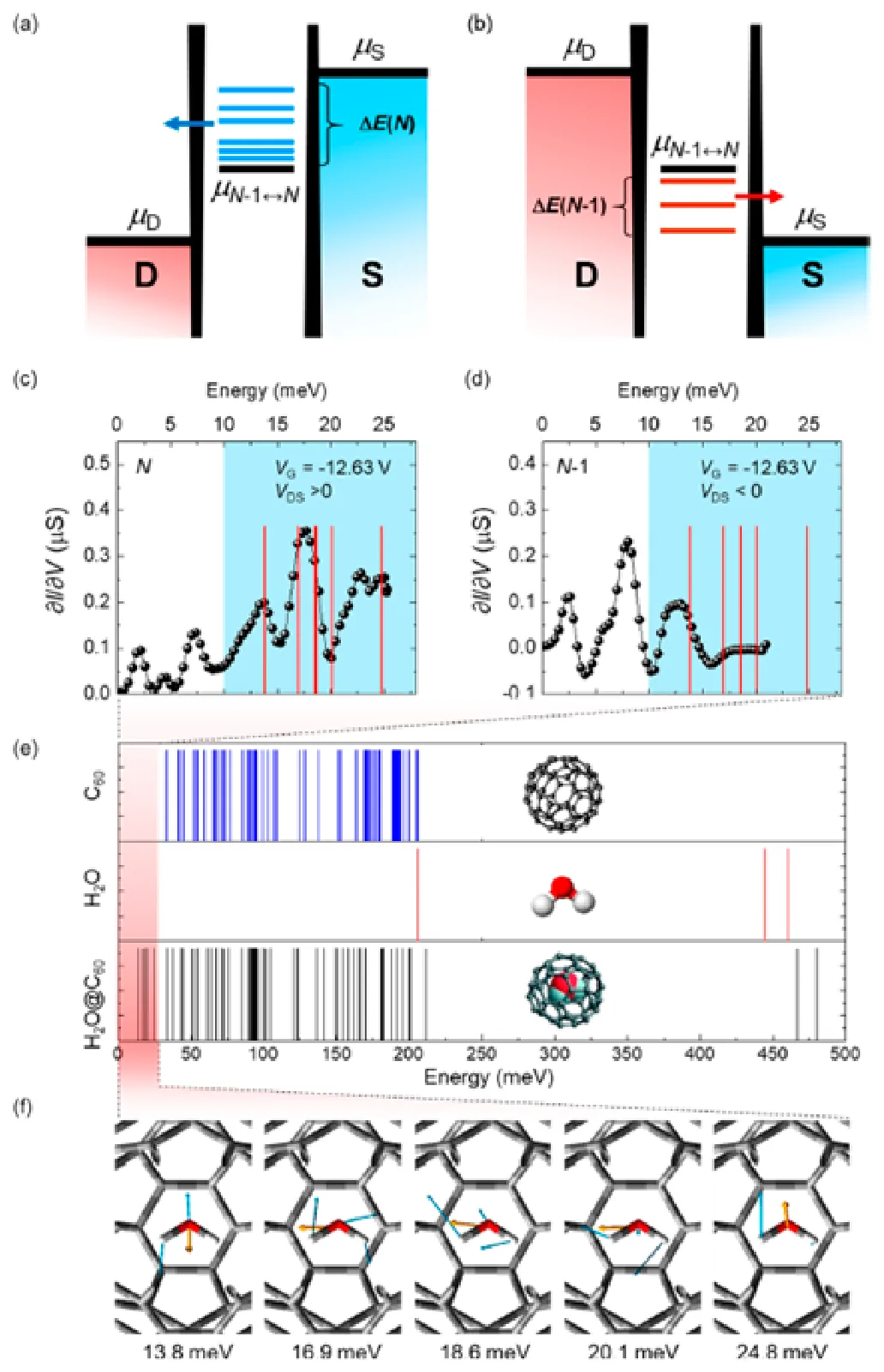
(**a**) Schematic illustration for tunnel conduction through the excited states for the N-electron state. (**b**) Similarly, tunneling process through the excited states for the (N − 1) electron state. (**c**) Differential conductance obtained as a function of positive VDS at VG = −12.63 V along the vertical dashed line in figure. By multiplying a conversion factor (0.67), VDS is converted to the excitation energy. (**d**) Differential conductance obtained as a function of negative VDS at VG = −12.63 V. By multiplying a similar conversion factor (0.58), VDS is converted to the excitation energy. (**e**) The calculated vibrational spectra for neutral C_60_, H_2_O, and H2O@C60 molecules. (**f**) Calculated five vibrational modes of the encapsulated water molecule in the C_60_ cage below 30 meV. The blue arrows show the displacement vectors, and the orange arrows represent the dipole derivative unit vectors Adapted from [88] licensed under CC BY 4.0.

Electron transport can probe the quantum rotational states of the confined molecule. Du and co-workers observed low-energy excitations in Coulomb-stability diagrams and THz photocurrent spectra of H_2_O@C_60_ single-molecule transistors. As shown in Figure 15, comparison with the calculated excitation energies supported the assignment of excitations below approximately 10 meV to the rotational states of para- and ortho-H_2_O [88]. Both nuclear-spin isomers were detected during measurements of a nominally single encapsulated molecule, implying fluctuations between ortho- and para-water faster than the experimental acquisition time of approximately one minute. The authors proposed that interactions with conducting electrons may accelerate spin-isomer interconversion. This interpretation extends the role of theory beyond equilibrium electronic structure: a complete model must eventually include inelastic electron tunneling, electron–rotation coupling, non-equilibrium populations, decoherence, and nuclear-spin conversion [88].

#### 4.4.4. Excess Electrons and Charged Endofullerenes

Calculations of diffuse excess-electron states provide another test of electrostatic shielding. Voora, Cederbaum, and Jordan predicted an s-type correlation-bound anion of C_60_ and compared it with He@C_60_, Ne@C_60_, and H_2_O@C_60_. For H_2_O@C_60_, the long-range dipole field contributed relatively little because the dipole of H_2_O was largely canceled by the induced response of the cage. Electron binding instead depended strongly on electron correlation and the fullerene polarization potential, including attractive contributions both inside and outside the cage [105]. Gas-phase electronic spectroscopy of H_2_O@C_60_^+^ and D_2_O@C_60_^+^, supported by DFT calculations, further demonstrated that electronic excitation of the charged fullerene cage can be accompanied by internal excitation of the encapsulated molecule. Although the principal electronic transitions were cage-centered, the rich vibronic structure revealed coupling between the changing cage charge distribution and the nuclear states of H_2_O or D_2_O [106].

### 4.5. Reactivity and Chemical Consequences of Encapsulation

The effect of internal water on the exohedral reactivity of C_60_ is strongly dependent on the reaction mechanism. When reactivity is governed mainly by the global fullerene π system and does not involve a strongly localized polar intermediate, encapsulation may exert only a minor influence. Reveles and co-workers calculated the thermodynamics and kinetics of the Diels–Alder reaction between 1,3-butadiene and the inequivalent bonds of C_60_ and H_2_O@C_60_. The reaction and activation energies were similar for the empty and water-containing cages and were largely independent of the orientation of H_2_O. The [6,6] bonds remained the preferred reaction sites. Born–Oppenheimer MD additionally showed rapid rotation of the internal water molecule at 300 K, which averages its small local polarization effect [107].

A substantially stronger effect emerges when the exohedral reaction produces a localized charged or strongly polar intermediate. Maroto and co-workers studied cis–trans isomerization in chiral fulleropyrrolidines derived from H_2_O@C_60_. Their DFT calculations showed that the encapsulated molecule can reorient and stabilize a zwitterionic or carbanionic intermediate through a directional interaction across the fullerene framework. This stabilization lowered the calculated barrier and contributed to the observed stereospecificity [85].

These studies establish a useful general principle: internal H_2_O has relatively little influence on reactions that do not generate significant localized charge separation, but it may become chemically important when the reaction coordinate creates a strong electrostatic perturbation. Computational studies of fullerene reactivity should therefore sample multiple guest orientations and should not assume that the water molecule remains fixed throughout the reaction [85,107].

The same effect is intensified in heterofullerenes. In H_2_O@C_59_N and particularly H_2_O@C_59_N^+^, the nitrogen dopant creates an asymmetric internal electrostatic field, strengthening water–cage coupling and influencing molecular dynamics and reactivity. Cage doping can therefore convert the weak, delocalized interaction characteristic of H_2_O@C_60_ into a more directional and chemically consequential host–guest interaction [75].

### 4.6. Beyond Pristine H_2_O@C_60_

#### 4.6.1. H_2_O@C_70_ and Cage-Shape Effects

**Figure 16:**
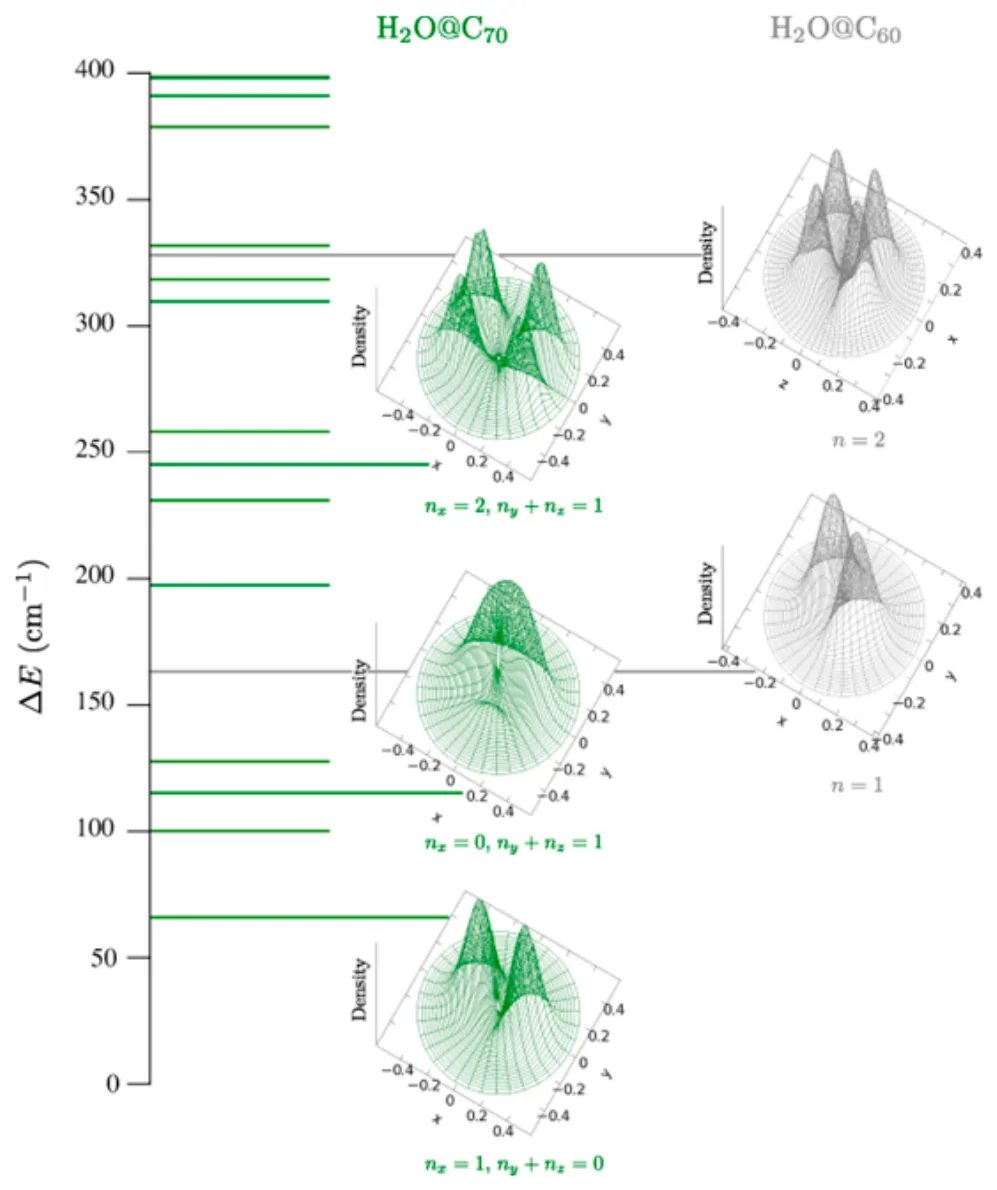
Comparison of the calculated translation–rotation energy levels and representative translational probability-density distributions for H_2_O confined in C_70_ (green) and C_60_ (gray). Reprinted with permission from [108].

C_70_ provides an important comparison because its elongated cavity lacks the nearly spherical symmetry of C_60_. Carrillo-Bohórquez, Valdés, and Prosmiti developed a full-dimensional MCTDH treatment of H_2_O@C_70_ that included translational, rotational, and vibrational degrees of freedom. The anisotropic host generated a more structured translation–rotation energy manifold and direction-dependent quantization of the water center-of-mass motion. As illustrated in Figure 16, the intrinsic anisotropy of the C_70_ cage lifts the near-degeneracy characteristic of H_2_O@C_60_, producing direction-dependent translational quantum states [108]. Comparison between H_2_O@C_60_ and H_2_O@C_70_ helps separate intrinsic cage anisotropy from symmetry breaking caused by crystal packing or deformation. In C_70_, the anisotropy is an inherent property of the isolated host, whereas in ideal C_60_ it arises primarily through weaker external or structural perturbations. These results indicate that cage size and shape provide an effective means of tuning guest localization, rotational freedom, and spectroscopic energy-level patterns [95,108].

#### 4.6.2. Coupled Endofullerene Assemblies

Calculations on H_2_O@C_60_ dimers and larger assemblies describe the transition from an isolated quantum rotor to a collective dipolar system. Twelve-dimensional and generalized multidimensional quantum models show that coupling between encapsulated dipoles can produce energy shifts, state mixing, and partial orientational ordering. However, these effects are weakened by screening through the fullerene cage and by quantum rotational delocalization of H_2_O. This competition explains why crystalline H_2_O@C_60_ exhibits dielectric activity but cannot be represented simply as a classical lattice of fixed dipoles. Each unit acts simultaneously as a permanent molecular dipole, a quantum rotor, and an electronically screened component of the fullerene crystal. Realistic bulk models must therefore include cage orientational order, state-dependent dipole coupling, nuclear-spin statistics, and temperature-dependent populations [100,101].

#### 4.6.3. Multiple Water Molecules and Confined Clusters

Several theoretical studies have explored fullerene systems containing multiple encapsulated water molecules, including (H_2_O)_3_@C_60_ and related confined water clusters. These models were primarily designed to investigate the physical limits of molecular confinement by examining hydrogen-bond formation, guest–guest interactions, internal pressure, cage deformation, and the evolution of the hydrogen-bond network with increasing water content [109,110,111,112]. In contrast to the experimentally established H_2_O@C_60_ system, where a single water molecule behaves as an almost freely rotating quantum rotor, confinement of multiple water molecules leads to the formation of intermolecular hydrogen bonds, reduced rotational mobility, and increasingly cooperative interactions between the encapsulated guests. At the same time, the resulting increase in internal pressure may induce measurable deformation of the fullerene cage and modify its mechanical properties [109,110,111,112].

Although these studies provide valuable insight into the fundamental physics of nanoconfined water, most of the proposed (H_2_O)_n_@C_60_ systems remain theoretical models. Consequently, they should be regarded as investigations of the limits of molecular confinement rather than as experimentally verified endofullerenes. Their conclusions therefore complement, rather than replace, the extensive experimental and theoretical understanding currently available for H_2_O@C_60_ and larger systems such as H_2_O@C_70_ [108].

Together, these computational studies demonstrate that H_2_O@C_60_ has evolved from a model system for nanoconfined water into a versatile platform for investigating quantum molecular dynamics, host–guest electrostatics, and confinement-controlled chemical phenomena. The remaining challenges and future research directions are discussed in the following section.

### 4.7. Current Limitations and Future Computational Directions

No single theoretical framework currently describes all relevant properties of H_2_O@C_60_ with uniform accuracy. Static density functional theory (DFT) is efficient for geometry optimization, charge-density analysis, and reaction pathways but remains sensitive to the choice of exchange–correlation functional and dispersion correction. Correlated wave-function methods provide more reliable interaction energies but remain computationally demanding for constructing multidimensional potential-energy surfaces. Six-dimensional quantum models successfully describe translation–rotation coupling but neglect the flexibility of the water molecule, whereas fully coupled nine-dimensional approaches provide a more complete description at substantially higher computational cost [94,95,96,97,98,100,101,102,113]. Because no single computational approach simultaneously captures all relevant aspects of H_2_O@C_60_, the principal theoretical methods, together with their applications, strengths, and limitations, are summarized in Table 3.

A quantitative comparison with experiment further illustrates the complementary strengths and limitations of these computational approaches (Table 4). High-level correlated calculations provide a reliable description of the host–guest interaction, with an interaction energy of approximately −14.26 kcal mol^−1^ and a zero-point-energy-corrected value of −14.11 kcal mol^−1^ reported for H_2_O@C_60_ [96], although a directly comparable experimental interaction energy is not currently available. For electrostatic properties, the agreement is particularly encouraging: ab initio molecular dynamics yielded a total dipole moment of approximately 0.55 D, arising from a water dipole of approximately 2.01 D partially canceled by an oppositely directed induced cage dipole of approximately 1.46 D [93], in close agreement with the experimentally derived effective dipole moment of approximately 0.50 D [76]. For translational motion, DF-LMP2 calculations predicted frustrated translational modes at approximately 90.5–94.3 cm^−1^ [96], compared with the experimentally observed excitation near 110 cm^−1^ [76]. Calculations also reproduce the experimentally observed tendency toward red-shifted intramolecular vibrations, although the predicted magnitude remains sensitive to the potential-energy surface and treatment of nuclear motion. Finally, the experimentally observed splitting of the ortho-H_2_O rotational ground state provides a particularly stringent test: idealized Ih-symmetric models do not generate this splitting, whereas calculations incorporating cage deformation, off-center displacement, crystal-field effects, or neighboring-cage electrostatics reproduce the observed symmetry reduction with varying quantitative accuracy [95,97,98,99,100]. These comparisons demonstrate that static electronic-structure methods are most useful for interaction energies, equilibrium structure, and polarization, whereas multidimensional quantum-dynamical approaches are required for a quantitative description of the coupled translational, rotational, and vibrational spectrum.

**Table 3:** Overview of computational studies on H_2_O@C_60_ and related endofullerene systems, including the principal computational methods, software, objectives, and representative applications.

Fullerene Derivative (Host)	Encapsulated Guest	Type of Calculation	Calculation Method	Computational Software	Aim of Study	Reference
C_60_	H_2_O (3 molecules)	GO, ESPA	ab initio RHF: STO-3G/3-21G/ 6-31G	Firefly	Structural characterization and dipole-moment analysis of a (H_2_O)_3_ cluster confined inside a C_60_ cage	[111]
C_60_	H_2_O (3 molecules)	GO, ESPA	ab initio RHF/6-31G(d,p) and DFT B3LYP5/6-31G(d,p)	Firefly	Evaluation of hydrogen-bond topology, cage distortion, and dipole moment of a (H_2_O)_3_ cluster confined in C_60_	[112]
C_60_	H_2_O	ESPA	MCTDH quantum dynamics (9D *n-mode* PES constructed from DFT-SAPT Lennard-Jones interactions and ab initio H_2_O monomer PES; TI/TD/BIR algorithms)	Heidelberg MCTDH package	Quantum characterization of coupled translational–rotational–vibrational states of H_2_O confined in C_60_	[94]
C_60_	H_2_O	ESPA	MCTDH quantum dynamics (9D *n-mode* PES including symmetry-breaking parameters from DFT-SAPT; BIR/IR procedures for energy-level computation)	Heidelberg MCTDH package	Analysis of symmetry breaking and rotational-level splitting induced by off-center H_2_O displacement in C_60_	[95]
C_60_	H_2_O	GO, ESPA	DF-LMP2/cc-pVTZ (geometry, frequencies), DLPNO-CCSD(T) (benchmark energies)	Molpro, ORCA, SNF	Benchmark evaluation of interaction energies, optimized geometries, and vibrational shifts in H_2_O@C_60_	[96]
C_60_	H_2_O (and H_2_, HF)	ESPA, TR, PT	DFT (LH12ct-SsIrPBE/cc-pVDZ)	NWChem, Custom variational quantum-dynamics code	Investigation of symmetry-breaking mechanisms and translational–rotational energy-level splitting in H_2_O@C_60_, with comparative analysis for H_2_@C_60_, NH_3_@C_60_, and HF@C_60_ complexes	[99]
C_60_ covalent dimer	H_2_O (2 molecules)	ESPA, TR	12D variational quantum dynamics using dipole–dipole coupling Hamiltonian	Custom variational TR-dynamics code (in-house)	Investigation of dipole-coupled translational–rotational states in the covalent (H_2_O@C_60_)_2_ dimer and evaluation of intermolecular coupling effects on TR-level structure and apparent symmetry breaking	[100]
C_60_	H_2_O (and NH_3_, CH_4_, HF)	GO, ESPA, SCRF, Kinetics (TST/Marcus)	DFT (BPW91/D95V; BPW91/6-311+G(d)), SMD (benzene, water)	Gaussian 09, DAMQT	Computational assessment of electronic structure, solvation effects, and free-radical scavenging activity of H_2_O@C_60_ and related CH_4_@C_60_, NH_3_@C_60_, HF@C_60_ systems compared to empty C_60_	[114]
C_60_	H_2_O (and NH_3_, H_2_, O_2_, O_3_)	GO, ESPA	DFT (B3LYP/6-31G(d))	Gaussian 03W	Investigation of structural stability, electronic characteristics, and inclusion energetics of H_2_O@C_60_, with comparative analysis for NH_3_, H_2_, O_2_, and O_3_	[115]
C_60_	H_2_O_n_ (n = 1, 3, 4)	GO, ESPA, MD	DFT (GGA-RPBE/DZP; norm-conserving pseudopotentials)	SIESTA, LAMMPS	Analysis of the impact of encapsulated water molecules (n = 1, 3, 4) on the mechanical properties, internal pressure, and bond stability of C_60_ under tensile, compressive, and hydrostatic strain	[110]
C_60_	H_2_O (and Li^+^, Na^+^, K^+^, Be^2+^, Mg^2+^, Ca^2+^)	GO, ESPA, TDDFT, SCRF	PM7 (pre-optimization), DFT (B3LYP/6-31G(2d,2p)), TDDFT (B3LYP/6-31G), PCM (toluene)	MOPAC, GAMESS-US	Analysis of structural, electronic, and optical properties of H_2_O@C_60_ compared with metal-ion C_60_ complexes, focusing on stability, reactivity, dipole moments, band gaps, and solvent effects	[116]
C_60_	H_2_O	GO, ESPA, MD, TRPMD	DFT (PBE-D3), ML-accelerated MD/TRPMD	VASP, Phonopy, LAMMPS, i-PI, MTP framework	Analysis of nuclear quantum effects on vibrational spectra, rotational relaxation, and O–H bond structure of H_2_O@C_60_ at cryogenic temperature	[103]
C_60_^+^	H_2_O (and D_2_O)	GO, ESPA	DFT (B3LYP/6-31++G(p,d), D3BJ, TDDFT single-point)	Gaussian 16	Analysis of gas-phase electronic spectra of nuclear-spin-isomer-separated H_2_O@C_60_^+^, with D_2_O@C_60_^+^ studied for isotopic comparison and assignment of electronic transitions	[106]
C_60_	H_2_O	ESPA	DFT (PBE-D3/DZVP-MOLOPT-SR; GTH pseudopotentials)	CP2K	Analysis of water–cage interactions and symmetry-breaking effects in H_2_O@C_60_ based on DFT-derived potential energy surfaces and S_6_-symmetry potential model	[97]
C_60_	H_2_O	GO, ESPA, MD	DFT (PBE/DZVP, all-electron, LCGTO)	deMon2k (with in-house BOMD module)	Investigation of thermodynamics, kinetics, and regioselectivity of the Diels–Alder reaction of 1,3-butadiene with H_2_O@C_60_ versus empty C_60_, and assessment of the effect of endohedral water orientation on reactivity	[107]
C_60_	H_2_O	ESPA, TR	6D TR quantum dynamics (LJ + electrostatic models)	In-house quantum-dynamics code (6D TR solver with INS module)	Quantum simulation of INS spectra of H_2_O@C_60_ and analysis of coupled translation–rotation dynamics and symmetry-breaking effects	[117]
C_60_	H_2_O	GO, ESPA, NEGF	DFT (GGA-PBE/DZP, norm-conserving pseudopotentials)	SIESTA, TRANSIESTA	Analysis of position- and dipole-dependent conductance modulation in H_2_O@C_60_ single-molecule junctions and demonstration of Faraday-shielding disappearance under bias	[118]
C_60_	H_2_O	GO, ESPA	DFT (PBE/3ζ)	Priroda	Prediction of pressure-induced inner-cavity reactivity of H_2_O@C_60_ leading to covalent endohedral derivative formation and analysis of structural and energetic changes under cage compression	[119]
C_60_	H_2_O	GO, ESPA, AIMD, BSSE	HF, MP2, DFT (BLYP, PBE, PBE-D, B3LYP, M06-2X/6-311G(2d,p))	Gaussian 09, CPMD	Analysis of dipole moment, interaction energy and polarization of H_2_O@C_60_	[93]
C_60_	H_2_O	GO, ESPA, EDA, BSSE	DFT (PBE0/6-311++G(d,p)), HF, MP2	Gaussian 03/09, GAMESS, AIM2000, AIMAll	Analysis of water–cage interactions, charge redistribution, vibrational shifts, and stabilization mechanisms in H_2_O@C_60_	[92]
C_60_	H_2_O	GO, MD, Vibrational analysis	ONIOM (B3LYP/cc-pVDZ for H_2_O; PM3 for C_60_)	Gaussian 03	Analysis of anharmonic vibrational dynamics and temperature-dependent IR line shapes of H_2_O@C_60_	[120]
C_60_	H_2_O	Vibrational analysis	DFT (B3LYP, PBE0/6-31G(d,p))	GAMESS, ABINIT	Comparison of vibrational coupling, mode mixing and atomic kinetic energies of H_2_O@C_60_ vs. isolated H_2_O and ice XI	[121]
C_60_	H_2_O	TR, Vibrational analysis	Fully coupled 9D translational–rotational–vibrational quantum dynamics	In-house quantum dynamics code	Characterization of coupled translation–rotation–vibrational states of H_2_O confined in C_60_	[102]
C_70_	H_2_O	TR, Vibrational analysis	Fully coupled 9D translational–rotational–vibrational quantum dynamics	Heidelberg MCTDH package	Full-dimensional quantum-dynamical characterization of the translational, rotational, and vibrational energy spectrum of H_2_O confined in C_70_, including the effects of the intrinsic anisotropy of the elongated fullerene cage	[108]
C_60_	(H_2_O)_n_ (n = 1–4)	GO, ESPA, Vibrational analysis, AIM	ab initio HF/6-31G, MP2/6-31G	Gaussian	Assessment of structural distortion, interaction energies, hydrogen-bonding patterns, and vibrational shifts in small water clusters confined within a C_60_ cage	[109]
C_60_	H_2_O	ESPA	DLPNO-CCSD(T)/def2-TZVP	ORCA	Benchmarking interaction energies and decomposition of host–guest stabilization contributions in H_2_O@C_60_ and related endohedral fullerenes	[122]
C_60_ dimer	H_2_O (mono- and di-encapsulated dimer)	GO, ESPA, NEGF	DFT (GGA-PBE; DZP for molecules, SZP for Au; norm-conserving pseudopotentials)	SIESTA; TRANSIESTA; Inelastica	Analysis of electron transport, conductance modulation, and negative differential resistance in C_60_-dimer junctions induced by encapsulated H_2_O position and dipole orientation	[123]
C_60_	H_2_O (and H_2_, HF)	ESPA (reactivity analysis)	DFT (M06-2X/6-311++G(d,p); OLYP/TZP)	Gaussian 09	Comparative analysis of how incarcerated polar molecules (H_2_O vs. HF) influence exohedral fullerene reactivity and stabilize zwitterionic intermediates in cis–trans isomerization reactions	[124]
C_60_	H_2_O	TR (6D variational quantum dynamics)	6D variational TR quantum dynamics (rigid H_2_O, rigid C_60_; LJ PES)	Custom variational TR-dynamics code (in-house)	Quantum characterization of translation–rotation eigenstates of H_2_O@C_60_ in an Ih-symmetric C_60_ cage (reference model)	[98]
C_60_ assemblies (clusters of n = 3–6 cages)	H_2_O (one per cage)	TR (many-body quantum dynamics)	Many-body variational TR quantum dynamics using a dipole–dipole coupling Hamiltonian; contracted basis constructed from monomer 6D TR eigenstates	Custom TR-dynamics implementation (in-house)	Quantum characterization of size-dependent translation–rotation eigenstates in H_2_O@C_60_ assemblies and analysis of collective dipolar coupling effects	[101]
C_60_	H_2_O	GO, ESPA, MD (rotational dynamics, vibrational analysis)	DFT (M06-2X/6-311G(2d,p)); classical MD (flexible H_2_O, LJ-based C–H_2_O interactions)	SIESTA, LAMMPS	Analysis of rotational dynamics, orientational potential energy surface, vibrational frequency shifts, and entropy of a single H_2_O molecule confined in a C_60_ cage	[104]
C_60_	H_2_O	ESPA, MD	Classical MD (rigid C_60_, SPC/E H_2_O); DFT (GGA-PBE)	LAMMPS (MD), DFT code not specified	Analysis of electric-field-driven transport of H_2_O@C_60_, including coupling between dipole orientation, rotational dynamics of encapsulated H_2_O, and translational motion of the fullerene cage	[125]
C_60_	H_2_O	ESPA	ab initio EOM-MP2; ab initio EOM-CCSD with diffuse augmented ANO basis sets (+s, +sp, +4s4p, +6s6p)	CFOUR	Investigation of a correlation-bound s-type anion state of H_2_O@C_60_ with comparative assessment of excess-electron binding and localization across endohedral C_60_ complexes containing different guests	[105]
C_59_N (aza [60]fullerene)	H_2_O	GO, ESPA	DFT (B3LYP/6-31G(d))	Gaussian	Comparison with H_2_O@C_60_ indicates modified cage–guest interactions induced by nitrogen doping	[75]
Exohedrally functionalized C_60_ (fulleropyrrolidine derivatives)	H_2_O	ESPA (reaction mechanism analysis)	DFT (M06-2X/6-311+G(d,p)//OLYP/TZP)	Gaussian	Encapsulated H_2_O stabilizes zwitterionic intermediates via directional hydrogen bonding, lowering the cis–trans isomerization barrier and enhancing enantiospecificity compared with empty-cage derivatives	[85]
C_60_	H_2_O	GO, ESPA	DFT (M06-2X/6-31G(d,p))	Gaussian	Static structural and electronic analysis	[86]
C_60_	H_2_O	GO, ESPA, PF	DFT (B3LYP/6-31G for C_60_ and H_2_O; B3LYP-D3/6-31G(d) for H_2_O@C_60_)	ND	Low-energy excitations (<10 meV) in transport and THz spectra assigned to quantum rotational states of encapsulated H_2_O; simultaneous observation of ortho- and para-water excitations indicates rapid spin-isomer interconversion driven by interaction with conducting electrons	[88]
C_60_	H_2_O (comparative: HF)	GO, ESPA, MD	DFT-D3 (PBE); ab initio molecular dynamics	VASP; CP2K	Off-center position inside C_60_ when adsorbed on metal surfaces, driven by adsorption-induced intra-cage electric fields; strong electronic screening by the fullerene cage despite substantial electrostatic polarization	[89]
C_60_^−^; C_59_N^−^	H_2_O	ESPA (electrostatic interaction analysis)	DFT (B3LYP/6-311++G(d,p))	Gaussian	Experimental trends rationalized by a DFT-based electrostatic model indicating net attractive guest–host interaction in H_2_O@C_60_^−^ and repulsive interaction in H_2_O@C_59_N^−^	[87]
C_60_	H_2_O	ESPA, electrostatic modeling	Qualitative DFT-based analysis of HOMO/LUMO polarization and charge redistribution induced by encapsulated H_2_O; classical capacitance model extracted from Coulomb-diamond features	ND	Water-induced polarization and a modified electrostatic environment within the junction	[90]
C_60_	H_2_O	ESPA, vibrational analysis	DFT (ωB97XD/6-31+G(2df,p))	Gaussian 16	Strong reduction in the effective dipole moment and IR intensity of encapsulated H_2_O attributed to polarization of the fullerene π-electron system	[84]

**Table 4:** Representative quantitative comparison between experimental observables and theoretical predictions for H_2_O@C_60_, illustrating the principal strengths and limitations of different computational approaches.

Observable	Representative Experiment	Representative Theoretical Prediction	Interpretation/Computational Performance
H_2_O–C_60_ interaction energy	No directly comparable experimental value available	−14.26 kcal mol^−1^; −14.11 kcal mol^−1^ including ZPE, DF-LMP2/DLPNO-CCSD(T) [96]	Correlated wave-function methods provide a useful benchmark for the depth of the host–guest potential, but direct experimental validation remains difficult.
Effective dipole moment	~0.50 D [76]	~0.55 D total; H_2_O ~2.01 D and oppositely directed cage contribution ~1.46 D [93]	Excellent quantitative agreement; calculations rationalize the strong electrostatic screening by polarization of the C_60_ cage.
Translational excitation	~110 cm^−1^ [76]	~90.5–94.3 cm^−1^, DF-LMP2 [96]	The correct energy scale is reproduced, although the calculated frequency is somewhat lower than the experiment.
Intramolecular vibrational shifts	Red shifts in the confined-water fundamentals relative to free H_2_O [42,76]	Red shifts of ~19.2, 110.4, and 123.8 cm^−1^ for bending, symmetric stretching, and antisymmetric stretching, respectively [96]	Theory reproduces the direction of the confinement effect; its magnitude is sensitive to the electronic-structure method and treatment of nuclear dynamics.
Rotational-state symmetry breaking	Ortho-H_2_O ground state split into singlet and doublet components [83]	Ideal Ih models do not produce the splitting; models including off-center displacement, cage deformation, and environmental electrostatics reproduce the symmetry reduction [95,97,98,99,100]	A sensitive benchmark showing that the crystal or local environment must be included beyond the ideal isolated-cage model.
Low-energy rotational excitations	Rotational excitations observed below ~10 meV in single-molecule transport and in INS/THz spectroscopy [39,40,83,88]	Variational and multidimensional quantum-dynamics calculations reproduce and assist assignment of the low-energy rotational manifold [94,98,102]	Quantum-dynamical methods are required; classical MD cannot reproduce discrete rotational quantization.

Classical molecular dynamics simulations access long timescales but neglect nuclear quantum effects, whereas path-integral approaches account for equilibrium quantum delocalization without fully describing real-time quantum coherence. Machine-learning potentials considerably extend the accessible simulation times but inherit the limitations of their reference electronic-structure methods and training datasets. Moreover, most current computational studies treat isolated H_2_O@C_60_ molecules and therefore neglect long-range crystal packing, substrate interactions, and electrode effects, all of which have been suggested experimentally and computationally to influence the electronic structure and symmetry of the confined water molecule [75,86,88,94,97,102,103].

A major unresolved challenge is therefore the development of globally accurate many-body potential-energy surfaces capable of simultaneously describing water flexibility, cage deformation, electronic polarization, and environmental perturbations arising from crystalline, surface-supported, or device configurations. Although pairwise Lennard–Jones models remain computationally attractive, they cannot fully reproduce non-additive induction effects, curvature-dependent dispersion interactions, charge redistribution, or the polarization response of the delocalized π-electron system. Likewise, high-level correlated calculations of realistic crystals or molecular junctions remain computationally prohibitive. Future developments will likely rely on machine-learning potentials trained on coupled-cluster-quality data, explicitly polarizable interaction models, and quantum-dynamical simulations that incorporate crystal fields, surfaces, and electrode environments. Non-equilibrium transport calculations will also be required to describe electron-induced rotational excitation and ortho–para conversion, while combined path-integral and electronic-structure approaches may provide further insight into isotope effects in H_2_O@C_60_, D_2_O@C_60_, and H_2_^17^O@C_60_ [75,86,88,103].

Comparative investigations of different fullerene cages, isotopologues, heterofullerenes, charged species, and functionalized derivatives are expected to further clarify the relationship between confinement geometry and molecular dynamics. Whereas H_2_O@C_70_ provides a model system for studying the effects of intrinsic cage anisotropy, H_2_O@C_59_N and H_2_O@C_59_N^+^ demonstrate how localized electrostatic fields modify host–guest interactions. In parallel, single-molecule electronic devices have begun to reveal how quantized rotational states of confined water couple to electron transport through the surrounding fullerene cage [75,86,88,90,108].

Overall, theoretical studies consistently indicate that H_2_O@C_60_ cannot be adequately described as either a static inclusion complex or a freely rotating gas-phase water molecule. Instead, it represents a multidimensional quantum host–guest system in which weak intermolecular interactions generate experimentally observable changes in translation, rotation, vibration, nuclear-spin conversion, polarization, reactivity, and electron transport. The closest agreement between theory and experiment has been achieved by combining accurate electronic-structure calculations with nuclear quantum dynamics and experimentally validated interaction potentials, rather than by treating these phenomena independently. Continued integration of high-level electronic-structure methods, machine-learning potentials, nuclear quantum dynamics, and realistic environmental models is therefore expected to provide an increasingly comprehensive theoretical description of H_2_O@C_60_ and related endofullerenes.

## 5. Future Perspectives

The remarkable progress achieved for H_2_O@C_60_ has established this system as the benchmark for investigating isolated water under nanoscale confinement and has simultaneously stimulated the exploration of a broader family of molecular water endofullerenes. Larger fullerene cages, heterofullerenes, charged cages, functionalized derivatives, and isotopically labeled systems provide opportunities for systematically tuning host–guest interactions and exploring how confinement influences molecular dynamics [34,36,75,108].

Among these systems, H_2_O@C_70_ is particularly attractive because its elongated cavity introduces intrinsic anisotropy absent in nearly spherical C_60_, allowing the effects of cage geometry to be distinguished from those arising from crystal packing or external perturbations. Nitrogen-containing cages such as H_2_O@C_59_N and H_2_O@C_59_N^+^ introduce localized electrostatic fields that modify the potential-energy landscape experienced by the encapsulated water molecule, thereby providing model systems for investigating the relationship between confinement geometry, electronic structure, and molecular dynamics [74,75,108]. Continued advances in molecular-surgery methodologies are also expected to enable increasingly complex guest molecules, multiple encapsulated species, and functionalized fullerene frameworks, providing valuable platforms for investigating confined hydrogen-bond networks, cooperative molecular dynamics, and electronically tunable host–guest interactions beyond the single-molecule limit represented by H_2_O@C_60_ [34,36,74,75,108].

Several experimentally established properties of H_2_O@C_60_ may be relevant to discussions of possible future technological applications. Infrared, terahertz, and neutron-scattering experiments have directly resolved quantized rotational and translational excitations of the confined water molecule, while time-domain terahertz spectroscopy has demonstrated long-lived rotational coherence at low temperature [39,40,76,83]. Dielectric measurements have shown that the encapsulated molecular dipoles remain electrically detectable despite substantial electrostatic screening by the fullerene cage [41,78]. Surface-sensitive measurements further demonstrated that the position of the confined H_2_O molecule can respond to adsorption-induced intrafullerene electric fields [89]. At the single-molecule level, electronic-junction and transistor experiments have demonstrated charge transport through H_2_O@C_60_, gate-dependent electrostatic effects, and low-energy transport features associated with rotational excitations of the encapsulated molecule [86,88,90]. These observations demonstrate that the internal quantum dynamics of H_2_O@C_60_ can be experimentally detected and that selected observables are sensitive to the surrounding environment, although this should not yet be interpreted as controlled operation of a functional device.

By contrast, the use of H_2_O@C_60_ in quantum sensing, spin-based information processing, electrically controlled molecular rotors, or practical molecular-electronic devices remains prospective. Although rotational states, nuclear-spin isomerism, dielectric response, and electronic coupling have been observed experimentally, reproducible preparation, manipulation, and readout of selected quantum states have not yet been demonstrated at the level required for functional technologies. In particular, the strong electrostatic screening produced by the C_60_ cage, while advantageous for isolating the confined molecule from environmental perturbations, simultaneously limits direct external control of its dipole orientation and rotational or nuclear-spin state. H_2_O@C_60_ should therefore currently be regarded primarily as a highly controlled experimental platform for studying quantum molecular motion and host–guest coupling rather than as an immediately deployable quantum or molecular-electronic component.

Any future translation of these proof-of-principle observations into functional devices would require substantial progress on several fronts. External manipulation of the confined molecule must become sufficiently selective to control rotational, orientational, or nuclear-spin states without destroying the weakly perturbed confinement environment. State readout must be made more robust and reproducible, particularly in single-molecule electronic architectures where junction geometry strongly influences the measured response. Device-to-device variability, long-term stability, and reproducibility of molecular junctions remain important practical limitations, while scalable fabrication and integration of individually addressable endofullerenes into larger device architectures have yet to be established. Synthetic challenges also remain significant: molecular-surgery routes must provide higher overall yields, simpler procedures, lower production costs, and reliable access to isotopically labeled or chemically modified endofullerenes in sufficient quantities and purity. From the theoretical perspective, realistic descriptions of future devices will require models that simultaneously include nuclear quantum dynamics, electronic polarization, crystal or substrate effects, and coupling to electrodes. Addressing these challenges would be necessary before it can be established whether the spectroscopic and quantum properties demonstrated for H_2_O@C_60_ can be translated into reproducible technological functionality.

## 6. Conclusions

The encapsulation of a single water molecule inside a fullerene cage has transformed H_2_O@C_60_ from a remarkable synthetic achievement into one of the best-characterized molecular host–guest systems. Advances in molecular-surgery methodologies, together with the rapid development of structural, spectroscopic, and computational investigations, have established H_2_O@C_60_ as a unique platform for exploring molecular behavior under nanoscale confinement [6,31,36,44]. A defining feature of H_2_O@C_60_ research has been the exceptional agreement achieved between experiment and theory. Structural studies have confirmed the remarkable rigidity of the fullerene cage, while complementary spectroscopic and computational investigations have revealed the intricate translational, rotational, vibrational, electronic, and nuclear-spin dynamics of the encapsulated water molecule. Collectively, these studies have provided one of the most comprehensive descriptions of a molecular host–guest system. Beyond its intrinsic chemical significance, H_2_O@C_60_ has emerged as a unique model for investigating the behavior of an isolated water molecule in the absence of hydrogen-bond networks. The system offers a valuable opportunity to examine quantum rotation, electrostatic screening, host–guest interactions, and confinement-induced phenomena under precisely defined nanoscale conditions, thereby providing an important benchmark for both experimental and theoretical studies of molecular confinement. Although practical applications remain at an early stage, ongoing developments in fullerene chemistry, molecular electronics, quantum spectroscopy, and computational modeling are expected to further broaden the scientific significance of molecular water endofullerenes. Future investigations involving larger fullerene cages, heterofullerenes, isotopically labeled derivatives, and electronically addressable molecular architectures are expected to provide new opportunities for understanding and ultimately controlling molecular behavior under extreme confinement. Overall, H_2_O@C_60_ represents considerably more than a single water molecule trapped inside a carbon cage. It has evolved into a multidisciplinary model system that bridges synthetic chemistry, spectroscopy, quantum dynamics, computational chemistry, condensed-matter physics, and molecular nanotechnology. The convergence of synthetic methodology, advanced spectroscopy, and quantum-mechanical modeling has provided not only fundamental insight into the physics and chemistry of confined molecules but has also established a robust foundation for the rational design of increasingly sophisticated endohedral fullerene systems and functional molecular nanomaterials.

## Figures and Tables

**Table 1:** Standardized comparison of the principal molecular-surgery strategies for the preparation of H_2_O@C_60_. Encapsulation efficiency refers to the fraction of water-loaded open-cage fullerene, whereas closure and overall yields refer to chemical conversion and isolated product yield, respectively. Because the reported overall yields are calculated from different starting materials, the corresponding yield basis is explicitly indicated.

Synthetic Strategy	H_2_O Loading Conditions	Encapsulation Efficiency	Cage-Closure Yield	Overall Yield	Scalability/Practical Considerations
Kurotobi–Murata [36]	High-pressure loading, 9000 atm	Essentially quantitative	Final cage-reclosure step: ~29%	~5% from C_60_ over the original six-step route	Produced bulk material, but requires specialized very-high-pressure equipment and a multistep procedure
Krachmalnicoff–Levitt–Whitby [44]	H_2_O loading in 1-chloronaphthalene at 100 °C under atmospheric pressure	~78%	Optimized partial closure: ~69%; final Diels–Alder/retro-Diels–Alder closure: ~90%; purified closure sequence: ~72%	~15% from C_60_	Explicitly developed as a scalable route; avoids high-pressure water loading and markedly facilitates preparative synthesis
Hashikawa et al. [73]	9000 atm, 160 °C, 24 h using redesigned open-cage precursor 2f	~98%	H_2_O@3f formation up to ~80%; final pyrolytic closure up to ~87%	~70% from open-cage precursor 2f	Demonstrated on a practical scale (160 mg precursor); highly efficient, but still requires very-high-pressure loading equipment

**Table 2:** Synthesis and experimental characterization of H_2_O@C_60_ and related water-containing fullerene structures.

Fullerene Derivative (Host)	Encapsulated Guest	Synthesis/Preparation Method	Analytical Techniques	Key Observations/Findings	Limitations	Reference
C_60_ open-cage derivatives (2a–2c)	H_2_O (and D_2_O); reversible inclusion in an open-cage derivative	Sequential cage-scission and ring-expansion reactions of a ketolactam-derived open-cage C_60_ precursor afforded derivatives with enlarged orifices capable of reversibly encapsulating H_2_O or D_2_O	^1^H NMR spectroscopy and mass spectrometry of H_2_O@2a (and D_2_O@2a) adduct	Singlet ^1^H NMR near −10 ppm, consistent with water located within the open cavity; disappearance upon dehydration, indicating reversible inclusion. MS signals corresponding to H_2_O@2a (and D_2_O@2a), with mass shifts matching a single incorporated H_2_O (and D_2_O) molecule	Reversible inclusion limited by the open-cage architecture; water accommodated only while the orifice remains accessible. Complex stability dependent on solvent and hydration state; the guest was not permanently trapped because the cage remained open, and gradual thermal release or guest exchange could occur	[72]
C_60_	H_2_O (with HDO and D_2_O studied for isotopic comparison)	Original Kurotobi-Murata molecular-surgery method: high-pressure insertion of H_2_O into an open-cage precursor, followed by phosphite-mediated closure and HPLC purification	^1^H and ^13^C NMR spectroscopy, APCI-MS, single-crystal XRD (H_2_O@C_60_·(NiOEP)_2_), UV–vis spectroscopy, and cyclic voltammetry	First successful bulk synthesis of H_2_O@C_60_ via molecular surgery; quantitative water encapsulation under high pressure; X-ray diffraction confirmed encapsulation and indicated preferential guest orientation with minimal cage distortion; strong upfield ^1^H NMR shift (−4.81 ppm); high thermal and electrochemical stability	Requires very high pressure (9000 atm); although water encapsulation is essentially quantitative, the final cage-reclosure step was obtained in approximately 29% yield, and the original six-step synthesis afforded H_2_O@C_60_ in approximately 5% overall yield from C_60_; specialized high-pressure equipment and a multistep procedure limit practical scalability	[36]
C_60_	H_2_O (also D_2_O and H_2_)	Modified molecular-surgery route enabling low-pressure incorporation of H_2_O (or D_2_O) into an open-cage precursor, followed by phosphine-mediated cage closure and Diels–Alder/retro-Diels–Alder restoration of the fullerene framework; adapted for H_2_ encapsulation under moderate pressure	^1^H NMR; ^13^C NMR; ESI–MS; chromatographic purification	High-yield and scalable synthesis of H_2_O@C_60_ under mild conditions without extreme pressure; identification of a more reactive partial-closure intermediate improving closure efficiency; protocol transferable to D_2_O@C_60_ and to H_2_@C_60_ using analogous reaction sequences	Formation of competing partially closed by-products depending on reducing agent; closure yields sensitive to reaction conditions; H_2_@C_60_ requires processing under hydrogen pressure to prevent guest loss	[44]
C_59_N (azafullerene)	H_2_O (also H_2_)	Molecular-surgery synthesis of endohedral aza [60]fullerenes. H_2_O@C_59_N obtained by two complementary routes: (i) conversion from H_2_O@C_60_ via azafullerene formation and (ii) direct molecular-surgery synthesis from an open-cage C_59_N precursor with subsequent guest insertion and cage closure	^1^H and ^13^C NMR spectroscopy, variable-temperature NMR, spin–lattice and spin–spin relaxation measurements (T_1_, T_2_), APCI-MS, HPLC	First successful synthesis of H_2_O@C_59_N and H_2_@C_59_N; encapsulation preserved during azafullerene formation. Variable-temperature NMR and relaxation measurements revealed stronger guest–host interactions and more restricted rotational dynamics than in H_2_O@C_60_	Multistep molecular-surgery synthesis with relatively low overall yield; characterization relied primarily on NMR and mass spectrometry without independent crystallographic confirmation of the encapsulated structure	[75]
C_59_N^+^ (azafullerenyl cation)	H_2_O (and H_2_)	C_59_N^+^ generated in high yield by protonation of (C_59_N)_2_ with triflic acid, followed by dissociation to the monomeric azafullerenyl cation; subsequent treatment with H_2_O or H_2_ afforded endohedral C_59_N^+^ complexes, which were isolated and purified chromatographically	^1^H NMR, ^13^C NMR, variable-temperature NMR relaxation analysis, ESI–MS	Successful preparation of stable H_2_O@C_59_N^+^ and H_2_@C_59_N^+^; pronounced NMR shifts and relaxation behavior indicating strong guest–host interactions and restricted internal motion relative to neutral H_2_O@C_60_	Dynamic behavior inferred mainly from NMR relaxation without independent structural probes; cationic complexes sensitive to moisture and strong acids; stability of C_59_N^+^ limits extended thermal studies	[74]
C_60_	H_2_O (H_2_^17^O used for isotopic resolution)	H_2_O@C_60_ prepared previously by the Kurotobi–Murata molecular-surgery route; chromatographic purification and multi-step handling; ^17^O-enriched water (≈90%) used to enable heteronuclear NMR analysis	Variable-temperature ^1^H NMR; ^17^O NMR; linewidth and lineshape analysis; T_1_ relaxation measurements; Liouville-space modeling; spectral simulations	Resolution of a well-defined ^1^H sextet arising from ^1^H–^17^O J-coupling; linewidths explained by scalar relaxation of the second kind; rotational correlation time consistent with rapid isotropic reorientation of encapsulated H_2_O	Study confined to solution NMR without structural confirmation by diffraction; rotational model derived phenomenologically	[77]
C_60_	H_2_O	High-pressure incorporation of H_2_O into a redesigned open-cage precursor (2f) bearing a phenyl–imine orifice, followed by phosphine-mediated cage closure and thermal restoration of the fullerene framework to yield H_2_O@C_60_. The optimized orifice design improved intermediate stability and overall synthetic efficiency	^1^H NMR, APCI-MS, single-crystal XRD	Optimized orifice design enabled nearly quantitative water encapsulation (up to 98%) and increased the overall yield of H_2_O@C_60_ to ~70%, substantially improving earlier molecular-surgery protocols. XRD confirmed a centrally located water molecule within the intact fullerene cage	High-pressure conditions required for insertion; closure efficiency strongly dependent on phosphine identity; thermal conversion sensitive to impurities, with partially closed intermediates prone to decomposition unless purified prior to pyrolysis	[73]
C_60_	H_2_O	H_2_O@C_60_ prepared previously by the Kurotobi–Murata molecular-surgery method; nitroxide-functionalized C_60_ derivatives synthesized for paramagnetic relaxation studies; TEMPO added as an external paramagnetic probe	^1^H NMR spectroscopy; spin–lattice relaxation time (T_1_) measurements; temperature-dependent studies in solvents of varying viscosity and polarity	Spin–rotation-dominated proton spin–lattice relaxation of encapsulated H_2_O; weak coupling to the external environment demonstrated by minimal effects of solvent viscosity and polarity; comparable paramagnetic relaxation enhancements for H_2_O@C_60_ and H_2_@C_60_, consistent with through-space magnetic interactions	No direct structural characterization of guest position or cage distortion; dynamical parameters inferred indirectly from relaxation behavior	[79]
C_60_	H_2_O	H_2_O@C_60_ synthesized via the Kurotobi–Murata molecular-surgery route; purified by column chromatography followed by multi-stage high-vacuum sublimation up to 550 °C to remove protonated impurities and obtain material suitable for solid-state NMR	^1^H static and MAS NMR; double-quantum-filtered dipolar recoupling (DQfDR); numerical spectral simulations (SIMPSON) for fitting of dipolar and CSA tensors	Residual ^1^H–^1^H dipolar coupling and proton chemical-shift anisotropy (CSA) confirmed quantized rotational dynamics of encapsulated H_2_O. NMR tensor analysis indicated approximately perpendicular CSA and dipolar tensors and suggested cage distortion or electronic polarization rather than partial water alignment	Cage distortion inferred indirectly from NMR tensor analysis without independent structural validation (e.g., diffraction); CSA sign not uniquely determinable; study restricted to NMR-based methods	[80]
C_60_ and selected open-cage derivatives	H_2_O (and H_2_, HD, NH_3_, CH_4_ for comparison)	Preparation of open-cage fullerene derivatives enabling reversible inclusion of small molecules; encapsulation achieved prior to final cage closure or retained only while the orifice remained accessible; para-H_2_ enrichment performed using zeolite adsorption combined with liquid-O_2_ treatment; nitroxide-functionalized C_60_ derivatives employed for magnetic-switching studies	^1^H NMR, ^17^O NMR, FIR, INS, MAS-NMR, ENDOR	Summary of spectroscopic evidence for ortho- and para-H_2_O spin isomers, symmetry breaking, and rotational-level splitting in H_2_O@C_60_. Comparative analysis of H_2_O and other small endohedral guests highlights the influence of nanoscale confinement on molecular dynamics and nuclear-spin behavior	Encapsulation reversible and restricted to open-cage architectures; measurements largely limited to cryogenic temperatures; multiple guest species studied without isolation of single, structurally defined host–guest complexes	[81]
C_60_	H_2_O	H_2_O@C_60_ prepared previously by the Kurotobi–Murata molecular-surgery route; crystalline samples obtained by slow solvent evaporation and used directly for NMR experiments	Low-temperature solid-state ^1^H NMR; ^17^O NMR; MAS NMR; variable-temperature NMR; NMR relaxation measurements (T_1_, T_2_); nuclear-spin isomer population analysis	Direct observation of ortho- and para-H_2_O spin isomers; temperature-dependent spin-isomer populations and NMR relaxation measurements revealed quantized rotational motion and confinement-induced symmetry breaking inside C_60_.	No new synthesis developed; absence of complementary structural probes such as XRD or neutron diffraction; experimental sensitivity limited to NMR-active spin isomers	[22]
C_60_	H_2_O	H_2_O@C_60_ prepared previously by molecular-surgery techniques; solutions prepared in the nematic liquid crystal MBBA	^1^H and ^13^C NMR spectroscopy; temperature-dependent measurements in nematic and isotropic phases; analysis of residual dipolar couplings (RDC) and residual chemical shift anisotropy (RCSA)	Temperature-dependent residual dipolar couplings revealed partial orientational ordering of encapsulated H_2_O in a nematic liquid-crystal environment, consistent with weakly distorted and partially aligned C_60_ cages	No independent structural characterization of cage distortion; conclusions based on NMR anisotropic interactions in an external anisotropic medium	[38]
C_60_	H_2_O (^17^O-enriched)	H_2_O@C_60_ prepared previously by molecular surgery (^17^O-enriched water); dissolved in MBBA nematic liquid crystal	^1^H and ^17^O NMR spectroscopy; temperature-dependent measurements; analysis of residual dipolar coupling (RDC), residual quadrupolar coupling (RQC), and residual chemical shift anisotropy (RCSA)	Partial alignment of encapsulated H_2_O in a nematic liquid-crystal matrix; determination of molecular alignment order parameters; preferred orientation with the H–O–H molecular plane perpendicular to the liquid-crystal alignment axis	No independent structural probe; alignment model inferred from anisotropic NMR interactions	[82]
C_60_	H_2_O	H_2_O@C_60_ prepared previously using the Kurotobi–Murata molecular-surgery method; crystalline samples obtained by solvent evaporation and used directly for neutron spectroscopy	Inelastic neutron scattering (INS); temperature-dependent neutron spectroscopy (5–300 K)	INS resolved rotational transitions of ortho- and para-H_2_O and monitored slow nuclear-spin conversion. The threefold-degenerate ground rotational state of ortho-H_2_O was split into a doublet and a singlet, indicating symmetry breaking in the local cage environment	No new synthesis; no structural refinement by XRD; crystalline samples obtained under conditions optimized for INS rather than structural refinement; limited sensitivity to weak vibrational modes	[83]
C_60_	H_2_O	H_2_O@C_60_ prepared previously by the Kurotobi–Murata molecular-surgery method; solid mixtures of H_2_O@C_60_ and C_60_ pressed into pellets	Far-infrared and mid-infrared absorption spectroscopy; temperature-dependent measurements (5–45 K); analysis of ortho–para conversion dynamics	Experimental identification of rotational, translational, vibrational, and rovibrational modes of encapsulated H_2_O; observation of red-shifted vibrational frequencies and crystal-field-induced splitting of rotational states	Interpretation relies on spectroscopic modeling; no independent structural or diffraction-based determination of cage distortion or guest position	[76]
C_60_	H_2_O	H_2_O@C_60_ obtained by molecular surgery (Kurotobi–Murata protocol); material diluted with empty C_60_ to ca. 5% filling factor and pressed into pellets for THz measurements	Time-domain terahertz spectroscopy (THz-TDS, free-induction decay); temperature-dependent measurements down to 4 K	Observation of coherent rotational dynamics, ortho- and para-H_2_O transitions, and real-time ortho→para conversion. Rotational energy levels closely resemble those of free water, indicating weak guest–host interactions	No independent structural probe; interpretation of rotational coherence relies on spectroscopic analysis without dedicated theoretical modeling within the study	[40]
C_60_	H_2_O	H_2_O@C_60_ prepared previously by molecular-surgery methods; pressed pellets with an effective filling factor of approximately 5%	Terahertz time-domain spectroscopy (THz-TDS); Fourier-transform infrared (FT-IR) spectroscopy; temperature- and time-dependent measurements (6–300 K)	Identification of ortho- and para-H_2_O rotational transitions; observation of crystal-field-induced rotational-level splitting, low-frequency translational motion, and temperature-dependent ortho–para conversion	No independent structural characterization; spectral assignments based on phenomenological fitting without first-principles calculations	[39]
C_60_	H_2_O	Molecular surgery–based synthesis of H_2_O@C_60_ following the procedure originally developed by Murata and co-workers and subsequent literature reports; chromatographic purification	Temperature-dependent IR spectroscopy (10–300 K); synchrotron IR measurements; analysis of vibrational intensities	Strong reduction in the effective dipole moment and IR intensity of encapsulated H_2_O	The proposed lattice-phonon coupling mechanism remains to be experimentally verified	[84]
C_60_	H_2_O	Crystalline H_2_O@C_60_ prepared previously by the Kurotobi–Murata molecular-surgery method	Synchrotron X-ray diffraction with maximum entropy method (MEM) charge-density analysis; dielectric spectroscopy; temperature-dependent measurement	Experimental determination of the crystal structure and charge-density distribution of H_2_O@C_60_; observation of rotational motion persisting down to 8 K, an fcc→sc phase transition identical to empty C_60_, and Curie–Weiss dielectric behavior consistent with dipolar interactions	Hydrogen positions not directly resolved by X-ray diffraction; dipole moment estimated indirectly from dielectric data; no independent spectroscopic probe of rotational dynamics	[41]
Exohedrally functionalized C_60_ (fulleropyrrolidine derivatives)	H_2_O	Exohedral functionalization of preformed H_2_O@C_60_ via asymmetric 1,3-dipolar cycloaddition (no synthesis of H_2_O@C_60_ reported)	Chiral HPLC; NMR spectroscopy; kinetic cis–trans isomerization studies	Encapsulated H_2_O stabilizes zwitterionic intermediates via directional hydrogen bonding, lowering the cis–trans isomerization barrier and enhancing enantiospecificity compared with empty-cage derivatives	Used previously synthesized H_2_O@C_60_; no new endofullerene synthesis was reported. Accurate kinetic analysis was limited by the competing retro-Prato reaction	[85]
C_60_	H_2_O	Molecular surgery–derived H_2_O@C_60_ following established protocols; solution processing for single-molecule junction fabrication	Mechanically controllable break junction (MCBJ) measurements; conductance histograms; I–V characteristics of single-molecule junctions	Single-molecule conductance of H_2_O@C_60_ is comparable to that of empty C_60_, indicating effective electrostatic shielding of the encapsulated water molecule by the fullerene cage	Limited control over the orientation of H_2_O inside the cage	[86]
C_60_^−^; C_59_N^−^	H_2_O	H_2_O@C_60_ and H_2_O@C_59_N prepared previously by molecular-surgery methods; gas-phase anions generated by electrospray ionization and cryogenically cooled in an ion trap	High-resolution cryogenic photoelectron imaging spectroscopy; near-threshold electron affinity (EA) measurements; vibrationally resolved photoelectron spectra	Accurate electron affinities reveal opposite EA shifts in H_2_O@C_60_^−^ and H_2_O@C_59_N^−^; low-frequency features tentatively assigned to hindered H_2_O rotation	No independent structural determination of guest orientation	[87]
C_60_	H_2_O	H_2_O@C_60_ obtained previously by the Kurotobi–Murata molecular-surgery route; single molecules trapped between gold electrodes using an electrical break-junction technique to form single-molecule transistors (SMTs)	Single-molecule electron transport (Coulomb stability diagrams); differential conductance (dI/dV); terahertz (THz) photocurrent spectroscopy; cryogenic measurements	Low-energy transport and THz excitations assigned to quantum rotational states of encapsulated H_2_O, with evidence for rapid ortho–para interconversion	Single-molecule measurements limited to cryogenic temperature, lack of independent structural characterization	[88]
C_60_	H_2_O (comparative: HF)	H_2_O@C_60_ obtained previously via the Kurotobi–Murata molecular-surgery route; submonolayer films prepared by thermal sublimation on Ag(111) and Cu(111) surfaces under UHV conditions	Normal-incidence X-ray standing wave (NIXSW); STM; non-contact AFM; valence-band XPS; resonant photoemission	NIXSW directly locates the encapsulated H_2_O molecule at an off-center position inside C_60_ when adsorbed on metal surfaces. STM, AFM, and photoemission show no contribution of H_2_O to the frontier orbitals	No new synthesis of H_2_O@C_60_; structural information derived for surface-adsorbed systems only	[89]
C_60_	H_2_O	Molecular surgery to obtain endohedral H_2_O@C_60_; solution processing in toluene; fabrication of single-molecule junctions by feedback-controlled electromigration break junctions (FCEBJ)	Single-molecule transistor measurements at ≈2 K; differential conductance (dI/dV) maps; Coulomb blockade and Coulomb oscillations; gate-dependent transport analysis	Single-electron transport observed in H_2_O@C_60_ junctions with higher gate coupling than for empty C_60_, attributed to water-induced polarization and a modified electrostatic environment within the junction	Limited number of stable single-molecule junctions; restricted statistics due to junction-to-junction variability; indirect attribution of transport features to the encapsulated water molecule	[90]

## Data Availability

No new data were created or analyzed in this study. Data sharing is not applicable to this article.
